# The effects of peripheral hormone responses to exercise on adult hippocampal neurogenesis

**DOI:** 10.3389/fendo.2023.1202349

**Published:** 2023-11-24

**Authors:** Robert R. Kraemer, Bradley R. Kraemer

**Affiliations:** ^1^ Department of Kinesiology and Health Studies, Southeastern Louisiana University, Hammond, LA, United States; ^2^ Department of Biological Sciences, University of Alabama in Huntsville, Huntsville, AL, United States

**Keywords:** testosterone, insulin-like growth factor (IGF- I), exercise, neurogenesis, Brain-derived Neurotrophic Factor (BDNF), growth hormone, vascular endothelial growth factor (VEGF), estrogen

## Abstract

Over the last decade, a considerable amount of new data have revealed the beneficial effects of exercise on hippocampal neurogenesis and the maintenance or improvement of cognitive function. Investigations with animal models, as well as human studies, have yielded novel understanding of the mechanisms through which endocrine signaling can stimulate neurogenesis, as well as the effects of exercise on acute and/or chronic levels of these circulating hormones. Considering the effects of aging on the decline of specific endocrine factors that affect brain health, insights in this area of research are particularly important. In this review, we discuss how different forms of exercise influence the peripheral production of specific endocrine factors, with particular emphasis on brain-derived neurotrophic factor, growth hormone, insulin-like growth factor-1, ghrelin, estrogen, testosterone, irisin, vascular endothelial growth factor, erythropoietin, and cortisol. We also describe mechanisms through which these endocrine responses to exercise induce cellular changes that increase hippocampal neurogenesis and improve cognitive function.

## Introduction

In the adult mammalian brain, the production of new neurons is restricted to two distinct regions, the subventricular zone, which supplies new neurons for the olfactory bulb, and the subgranular zone of the dentate gyrus, which produces new granule cells in the hippocampus (1). Neurogenesis in the hippocampus is particularly important for learning and memory (2). Rates of hippocampal neurogenesis can be influenced by a variety of factors, among which exercise has been established as a potent stimulus (3, 4). Regulation of neurogenesis in response to exercise may significantly affect cognitive performance. For example, sedentary activity is associated with greater risk of cognitive decline, whereas a greater level of cardiorespiratory fitness is associated with a larger prefrontal cortex in older adults (5). Moreover, exercise reduces the decline in performance of independent living activities of those with early Alzheimer’s disease (4) and can change the structure and function of the hippocampus, a brain region that is critical for learning and memory (5, 6). Over the last decade, an abundance of other studies using animal models and human subjects have revealed positive effects of exercise in stimulating neurogenesis and reducing cognitive decline with aging. [e.g (3, 7–10)]. Thus, understanding the molecular interactions underlying these beneficial effects of exercise has become increasingly pertinent.

The effects of exercise on neurogenesis partially relate to changes in the brain in several neurotransmitter systems, including those for serotonin, dopamine, acetylcholine, and norepinephrine (for review, see (11)). However, our lab and others have also revealed a variety of peripheral endocrine responses to specific forms of exercise that are important for metabolism, tissue growth and repair, cardiovascular function, and other functions. [e.g (12–15)]. While many of these circulating hormones that respond to exercise stress, including estrogens and androgens (12, 13), somatotrophs (GH, IGF-1) (3, 13, 16, 17), VEGF (7) irisin (18, 19), and cortisol (20) are peripherally produced, emerging evidence indicates that these hormones and/or their effectors can cross the blood-brain barrier to profoundly influence neurogenesis and cognitive function. A number of these peripheral hormones can modulate neurogenesis during or after exercise by regulating activity of the neurotrophin, brain-derived neurotrophic factor, [e.g (21)], as well as through other pathways. Moreover, there is increasing interest in targeting these endocrine systems to reduce cognitive impairment associated with neurodegenerative diseases [e.g (22, 23)]. In the present review, we discuss changes in circulating hormone levels in response to different modes of exercise and the possible mechanisms through which these endocrine factors regulate neurogenesis.

## Brain-derived neurotrophic factor

Brain-derived neurotrophic factor (BDNF) is a neurotrophin that regulates diverse neural functions, including neurite growth, synaptic plasticity, neuronal differentiation, and cell survival (24). BDNF performs these functions through interactions with two different receptors. Activation of the tropomyosin-related kinase B (TrkB) receptor by BDNF stimulates signaling via the extracellular signal-regulated kinase (ERK), phospholipase Cƴ, and phosphoinositide 3-kinase (PI3K) pathways (25). BDNF can also activate the p75 Neurotrophin Receptor (p75^NTR^), a transmembrane protein that can promote c-Jun N-Terminal Kinase signaling, induce Nuclear Factor κB (NFκB) activity, and regulate Rho family members, among other functions (26). BDNF is produced at high levels in several brain regions, including the amygdala, cerebellum, cerebral cortex, and hippocampus (27) (28), and it is also synthesized by various non-neuronal cells, including vascular endothelial cells (29, 30), lymphocytes (31), and skeletal muscle cells (32, 33). There are two different pools of BDNF circulating in the blood, including platelet-bound BDNF and unbound plasma concentrations that can bind to TrkB or p75^NTR^ receptors (34, 35). Like other neurotrophins, BDNF is initially synthesized as a precursor, termed proBDNF, that can be proteolytically processed in the Golgi apparatus or in the extracellular mileu to yield mature BDNF. However, in certain physiological contexts, secreted proBDNF persists in its uncleaved form, which lacks affinity for Trk receptors and functions as a high-affinity ligand for the p75^NTR^-sortilin complex (26, 36).

Multiple lines of evidence have established BDNF as a key regulator of hippocampal neurogenesis. Several *in vitro* studies involving cultured neural precursor cells from the dentate gyrus or organotypic mice hippocampal slice cultures have revealed that BDNF induces cell proliferation, neuronal differentiation and neuronal survival (37–40). These findings have been corroborated by a variety of *in vivo* studies. For example, an analysis of heterozygous BDNF knockout mice revealed deficits in neurogenesis and reduced hippocampal volume (41), while another investigation demonstrated that intrahippocampal administration of BDNF increased the number of mature granule cells in the dentate gyrus (42). Beyond stimulating the genesis of new hippocampal granule cells, it is also well-established that BDNF promotes synaptogenesis, dendritic spine formation, and synaptic strengthening within the hippocampus (43). Thus, BDNF critically regulates the formation and modulation of hippocampal circuitry. Unfortunately, levels of BDNF decline in the periphery with age (44, 45), and aging-related loss of BDNF may occur in the hippocampus as well (46–48). Moreover, reductions in BDNF have been associated with decreased hippocampal volume and increased risk of dementia (44, 49, 50). Thus, studies elucidating strategies to enhance BDNF-induced neurogenesis and synaptic remodeling in the hippocampus may be of important therapeutic benefit. For example, 28-day infusions of BDNF into the entorhinal cortex, a region of the hippocampal formation that serves as an interface between the hippocampus and neocortex, were reported to enhance spatial memory in aged rats (51). Moreover, interventions that modulate BDNF signaling have been demonstrated to alter synapse loss or neuronal death in several rodent models of neurodegenerative disease (37), including models of Huntington’s disease (52), Parkinson’s disease (53), and Alzheimer’s disease (51).

Exercise is a powerful stimulus for increasing circulating BDNF. Transient increases in circulating BDNF occur in response to a variety of exercise modalities, including moderate-intensity aerobic exercise (54, 55), resistance training (54, 56), and high-intensity interval training (57, 58). Mechanisms through which exercise increases BDNF production may involve activation of Sirtuin-1 deacetylase by the metabolite lactate (59), stimulation of fibronectin type III domain-containing protein 5 (FNDC5) (60), production of beta-hydroxybutyrate (61), or induction of pro-BDNF cleavage by tissue-type plasminogen activator (tPA) (62), among other mechanisms. Additionally, BDNF production is regulated by multiple endocrine systems, as discussed in later sections of this article. Due to conflicting reports, the type of training modality that is most effective in enhancing circulating BDNF levels remains unclear. However, given that lactate concentrations stimulate hippocampal expression of BDNF, exercise regimens that are above an individual’s lactate threshold would be expected to induce greater circulating BDNF concentrations. Fittingly, multiple studies have indicated that the magnitude of increase in BDNF is intensity-dependent (63–65). Beyond the well-established effects of exercise in transiently increasing circulating BDNF, exercise training may also modestly enhance resting BDNF levels, though reports in this regard have been variable (34, 66). Tissue sources responsible for exercise-induced increases in circulating BDNF remain incompletely understood. While a small number of reports suggest that BDNF does not cross the blood-brain barrier (BBB) (67, 68), direct and indirect evidence from a greater number of studies indicate that BDNF bidirectionally traverses the BBB, as well as that circulating BDNF levels reflect brain levels (34, 69–73). Thus, exercise-induced increases in circulating BDNF may not only stem from peripheral sources such as release from vascular endothelial cells and platelets, but also from the brain (34, 72). Importantly, BDNF production is critical for exercise-induced cognitive benefits, since knockdown or antagonism of hippocampal BDNF prevents exercise-associated improvements in spatial reasoning and memory in rodents (66, 74, 75). Circulating BDNF likely serves a key role in these exercise-associated cognitive benefits, since peripheral administration of BDNF was sufficient to enhance hippocampal neurogenesis and hippocampal-dependent learning (73).

While a wealth of evidence has established BDNF as a positive regulator of hippocampal neurogenesis, the specific mechanisms through which BDNF increases the abundance of mature granule cells remain incompletely understood. One possibility is that BDNF stimulates proliferation of neural precursor cells (NPCs). Indeed, BDNF has been demonstrated to enhance NPC proliferation in neurosphere cultures (38, 76), and conditional disruption of BDNF signaling in hippocampal NPCs *in vivo* was reported to decrease proliferation (77). This ability of BDNF to stimulate NPC proliferation may involve activation of the transcription factor CREB via the TrkB-ERK pathway, since disruptions in hippocampal neurogenesis have been reported in animal models with genetically altered TrkB, ERK, or CREB signaling (77–79). CREB activation can stimulate cell proliferation through regulation of multiple targets, including CyclinD1, Replication Factor C3 (RFC3), proliferating cell nuclear antigen (PCNA), and JunD (80–85). However, the role for this pathway in promoting NPC proliferation has been contradicted by studies yielding evidence that BDNF, TrkB, and CREB do not positively impact NPC proliferation (86–90). Another possibility is that BDNF increases the number of granule cells by promoting NPC or granule cell survival. This hypothesis has been supported by multiple studies revealing decreased survival of hippocampal progenitor cells in mice with genetic depletion of BDNF (86, 87). This role for BDNF may be mediated through the TrkB-Akt pathway, since in injury models this pathway promotes survival of mature hippocampal neurons (91, 92). CREB, which can be activated by ERK or Akt, has also been demonstrated as necessary for the survival of newborn hippocampal neurons (89). However, the hypothesis that BDNF stimulates neurogenesis by facilitating the survival of hippocampal neurons has been contradicted by a report that deletion of *BDNF* in the central nervous system does not alter the survival of new hippocampal cells. Rather, the study indicated that BDNF promotes neurogenesis by facilitating the terminal differentiation of NPCs into mature granule cells (88). Altogether, the conflicting findings pertaining to the effects of BDNF on hippocampal NPCs may partially relate to developmental adaptions that may occur in animal models with genetic deletions. Thus, performing fate-mapping and thorough time-course assessments in models with temporally induced-genetic deletions may help to clarify the specific effects of BDNF-TrkB signaling on NPC proliferation, survival, and neuronal differentiation.

Of note, BDNF may not only regulate hippocampal neurogenesis through interaction with TrkB, but also through p75^NTR^. Multiple studies have revealed deficits in hippocampal neurogenesis in adult mice lacking p75^NTR^, suggesting that p75^NTR^ positively regulates neurogenesis (93–95). This promotion of neurogenesis by p75^NTR^ likely occurs through its crosstalk with TrkB, as p75^NTR^ has been demonstrated to augment TrkB signaling in hippocampal neurons (96). Interestingly, negative regulation of neurogenesis has also been reported in response to proBDNF, which binds to p75^NTR^ and sortilin but not TrkB (97, 98). Moreover, multiple types of exercise training have been demonstrated to enhance mature BDNF production in the hippocampus while concurrently stimulating in the brain the activity of tPA, a proteinase that cleaves proBDNF to yield mature BDNF (62, 99–102). Altogether, these findings suggest that exercise training can stimulate hippocampal neurogenesis by enhancing BDNF-TrkB-p75^NTR^ cascades while limiting activation of proBDNF-p75^NTR^-sortilin signaling.

## Growth hormone (somatotropin), insulin-like growth factor-1, and ghrelin

Growth hormone releasing hormone (GHRH) is a hypothalamic peptide hormone that is released during different forms of exercise. GHRH acts upon growth hormone releasing hormone receptor (GHRHR), a G protein-coupled receptor, activating the G_s_-PKA-cAMP signaling pathway and stimulating growth hormone (GH) release from the anterior pituitary (103, 104). Growth hormone (GH) is a somatotropin that is composed of a polypeptide chain with approximately 190 amino acid residues. It contains two disulphide bridges with four alpha helices and has two binding sites for two receptor molecules (105). GH is released in a pulsatile manner from the anterior pituitary and circulates to the liver, where it acts directly upon GH receptors (GHR) in hepatocytes. Activation of GHR induces Janus kinase (2)/signal transducers and activators of transcription 5 (JAK2/STAT5) signaling and stimulates production of insulin-like-growth factor 1 (IGF-1) from liver hepatocytes, as well as paracrine production of IGF-1 from other tissues (103). IGF-1 is a 70 amino-acid single chain peptide that has a molecular weight of 7.6 kDa and contains three disulphide bridges creating a tertiary structure important for optimal binding to the IGF-1R (106). Most IGF-1 polypeptides are transported by carrier proteins that determine the amount of IGF-1 available to different tissues (107). The actions of IGF-1 occur via IGF-1R, a membrane-bound receptor tyrosine kinase (RTK). IGF-1R activation can result in mitogen-activated protein kinase signaling, as well as activation of the PI3K-Akt signaling pathway, thereby promoting cell growth and maturation (108).

A variety of forms of exercise stimulate the GHRH/GH/IGF-1 axis, causing increased circulating concentrations of GH and IGF-1 (13, 16, 109, 110). Data from the Kraemer lab has revealed increases in circulating GH concentrations in response to three sets of four different resistance exercises, with GH averaging over an eight-fold increase during recovery (12). Additional research groups have reported strong GH increases in response to other resistance exercise protocols (111). GH response to anaerobic cycling has also been reported (112). We have also reported elevated circulating concentrations of both GH and IGF-1 in response to treadmill running at four progressively increased exercise intensities, with GH increase averaging approximately 5 times resting values while area under the curve values for IGF-1 revealed significantly higher levels over time (13). Others have reported GH increases in response to prolonged treadmill running at 60% VO_2_ max (113). In the aforementioned study of older men averaging 60.8y (54), increases in IGF-1 were observed in response to 30 min of both moderate intensity running and moderate intensity circuit weight training exercise. Collectively, these studies reveal that both aerobic exercise at moderate and high intensity as well as resistance exercise will stimulate acute increases in GH and IGF-1. In addition to acute responses to exercise, a systemic review of multiple aerobic and resistance training studies revealed that both forms of exercise training increase resting levels of GH and IGF-1 (114).

Secretion of GH from the pituitary gland is not only controlled by GHRH but also regulated by ghrelin, a 28 amino acid hunger hormone primarily produced in the stomach. There is some evidence that exercise training increases resting ghrelin levels (115–118), thus implicating ghrelin as a hormone that can potentially modulate GH-induced neurogenesis. However, for several of these studies, result interpretations are confounded by training-induced reductions in body weight (116–118), and conflicting studies have reported training-induced reductions in circulating ghrelin concentrations (119, 120). Additionally, circulating ghrelin concentrations do not increase in response to acute running or cycling (121, 122). Overall, due to limited and conflicting findings, further research is needed to clarify the relationship between various forms of exercise, ghrelin, and GH-IGF-1 signaling.

GH and IGF-1 are important regulators of neurogenesis and neuronal connectivity in the adult hippocampus. Both hormones can cross the blood-brain barrier (123, 124) and have specific receptors expressed in the central nervous system, including the hippocampus (125–127). Peripheral administration of GH has been demonstrated to enhance cellular proliferation in the dentate gyrus of healthy adult rats (128), as well as to increase the number of newborn neurons in the hippocampus in a rodent model of hypopituitarism (129). *In vitro* experiments from the aforementioned study suggest that GH increases hippocampal neurogenesis by stimulating NPC proliferation. However, GH has also been demonstrated to have a variety of neurotrophic actions in the central nervous system, including neuroprotection, axonal growth, and synaptogenesis (130, 131). Like GH, peripheral administration of IGF-1 has been demonstrated to enhance hippocampal neurogenesis (132). Evidence of the neurogenic potential of IGF-1 has also been corroborated by studies revealing that central infusion of IGF-1 increases the number of immature neurons in the dentate gyrus of rodent models of aging and traumatic brain injury (133, 134). Moreover, IGF-1 has been demonstrated to enhance synaptic complexity in the hippocampus, thereby suggesting that the hormone not only regulates hippocampal circuitry through modification of neuron abundance but also via regulation of neural connectivity (135). While the mechanisms through which GH and IGF-1 promote hippocampal neurogenesis remain incompletely understood, both hormones stimulate production of BDNF (136, 137). Additionally, IGF-1 has been shown to promote the proliferation of hippocampal NPCs and induction of pro-neural gene expression through a novel cascade involving activation of Sox2 by the Ras-related GTPase, RIT1 (138). IGF-1 signaling has also been linked to a variety of other signaling events, including suppression of proinflammatory cytokine signaling by IL-1β and TNF-α (137), activation of CREB (139), and modulation of glutamatergic neurotransmission (137). Interestingly, beyond stimulation of hippocampal neurogenesis by GH and IGF-1, hippocampal neurogenesis can also be directly stimulated by the GH secretagogue ghrelin. Ghrelin will bind to the GH secretagogue receptor GHS-R1a that has been identified in the rat brain, and *in vitro* experiments have revealed that ghrelin acts through the ERK1/2, PI3K/Akt, and STAT3 signaling pathways to stimulate neurogenesis cultured hippocampal NPCs (140). However, due to the aforementioned conflicting reports on ghrelin responses to various forms of exercise, the role of ghrelin in exercise-induced neurogenesis remains unclear.

In a process termed somatopause, there is a considerable and progressive reduction in circulating GH and IGF-1 with aging (141). Additionally, multiple human studies have indicated that higher levels of circulating GH and IGF-1 correlate with improved cognitive performance (142, 143). Thus, the GHRH-GH-IGF-1 axis may represent a promising target for therapeutic interventions to improve cognitive deficits. Indeed, administration of GH has been demonstrated to improve cognitive deficits associated with cortical impact in rats (136), as well as reduce tissue loss and enhance memory function in a mouse model of stroke (144). Moreover, while the cognitive benefits of exercise have been well-established (145), emerging evidence suggests a key role for GH-induced neurogenesis in exercise-induced cognitive improvement. For example, a recent investigation by Blackmore et al. revealed that GH signaling and neurogenesis are necessary for exercise-induced improvement in hippocampal-dependent spatial learning in aged mice (146).

In summary, utilizing different forms of acute exercise at the proper exercise intensity could be helpful in promoting neurogenesis in patients with neurodegeneration via increases in GH and IGF-1. In addition, exercise training may be helpful in maintaining hippocampal function by increasing resting circulating ghrelin levels. (147).

## Estradiol

17ß estradiol (Estrogen/E_2_) is a steroid hormone that exerts diverse actions after being produced in the ovaries, liver, heart, muscle, bone and brain (148). The production of E_2_ occurs via activation of the hypothalamic-pituitary-gonadal axis (149). Gonadotropin-releasing hormone (GnRH), a 10-amino acid peptide, is secreted by hypothalamic neurons into the median eminence and is then transported through the hypophyseal portal system to act upon GnRH receptors in pituitary gonadotrope cells (150). This signals production and secretion of luteinizing hormone (LH) and follicle-stimulating hormone (FSH) (151). Circulating LH and testosterone act on receptors in the gonads, stimulating release of the sex steroids, androgens and estrogens (151). Circulating E_2_ can cross the blood-brain barrier and bind to ERα, ERβ, and the G protein-coupled estrogen receptor 1 (GPER1) that are expressed in in both genders and found in multiple areas of the brain, including the hippocampus (152). A majority of the actions of E_2_ are thought to be induced through ERα and ERβ receptors (153). Binding of E_2_ to ERα and ERβ stimulates formation of a receptor-ligand complex that dimerizes and translocates to the nucleus, where it binds to estrogen response elements on DNA (154) and regulates gene transcription (155). There are also estrogen receptors in the cell membrane, and their binding induces quick, non-genomic effects such as changing cell permeability and stimulating 2^nd^ messenger cascades (156).

Changes in circulating E_2_ have been reported to respond to a variety of forms of exercise. Bunt et al. revealed that 60 min of treadmill running at 60% VO_2_max in trained and untrained male and female runners elicited significant increases in E_2_ in all subjects (157). Similarly, Gray et al. reported a 45% increase in E_2_ in young men after an average of 15.6 one-minute treadmill runs (158). Our lab has reported increases in E_2_ in young women in response to three sets of four resistance exercises with greater increases during the luteal than the follicular phase of the menstrual cycle (17). Interestingly, in the aforementioned studies, elevated levels of E_2_ after exercise correlated with increases in GH (158). Moreover, hormone replacement therapy (HRT) in post-menopausal women has been demonstrated to induce greater GH responses to 30 min of treadmill exercise compared to those not taking HRT (159). Thus, higher E_2_ concentrations with exercise can increase GH levels and may thereby potentially exert positive effects on adult neurogenesis.

Accumulating evidence also suggests that E_2_ can function as a direct, positive regulator of adult neurogenesis. The E_2_ receptors ERα and ERβ are expressed in the hippocampal NPCs (160) and multiple studies have indicated that E_2_ signaling enhances NPC proliferation. For example, E_2_ treatment was demonstrated to induce proliferation in NPC cultures of both embryonic rat and human cell-line origin (161–163). In a separate study, chronic estradiol treatment of spontaneous hypertensive rats resulted in reduced blood pressure, increased neurogenesis in the hippocampus, and increased BDNF RNA and protein expression in the dentate gyrus (164). Considering that the BDNF gene contains an estrogen-sensitive response element (ERE), the upregulation of BDNF expression may represent one mechanism through which E2 stimulates NPC proliferation (165). However, the effects of E2 on NPC proliferation have also been attributed to ERß-mediated activation of ERK and subsequent centrosome amplification (163).

Fitting with the potential role of E_2_ in regulating hippocampal neurogenesis, the hormone has also been demonstrated to confer cognitive benefits in specific physiological contexts. For example, E_2_ hormone therapy has been shown to be more protective of cognition in women with greater risk for Alzheimer’s Disease who continue to use it up to two years following menopause onset (166). Given that E_2_ has been shown to protect neural tissue (167, 168) more research is needed to establish the optimal exercise protocols for enhancing the effectiveness of medication in patients taking hormone replacement therapy to prevent cognitive decline following menopause.

## Testosterone

The hypothalamic-pituitary-gonadal axis is responsible for regulating testosterone production. Secretion of gonadotropin-releasing hormone (GnRH) by the hypothalamus induces release of luteinizing hormone (LH) and follicle stimulating hormone (FSH) from the anterior pituitary (169). LH stimulates production and release of testosterone from the testes and adrenal glands in men (170) and from ovaries, adrenal glands and peripheral tissues in women (171). In a variety of tissue types, testosterone can be metabolized via the enzyme 5α-reductase to dihydrotestosterone (DHT) (172). Testosterone and DHT function as androgens that bind to nuclear receptor subfamily 3, group C, member 4 (NR3C4)(173). Upon activation in the cytoplasm, the androgen receptor translocates to the nucleus, where it functions as a transcription factor (174). Testosterone circulating in men will be converted into both active metabolites, E2 and DHT, that will mediate some testosterone action in the target tissues (175).

Multiple studies have revealed acute changes in testosterone levels in response to different forms of exercise in males and females. Strenuous intermittent exercise consisting of treadmill running at 60%, 75%, 90% and 100% of VO_2max_ was shown to increase circulating testosterone levels in young males (176). Moreover, testosterone levels were reported to significantly increase in young women athletes in response to a discontinuous treadmill test to exhaustion, with 4- and 7-week training resulting in higher testosterone responses than 1-week training (177). Both concentric as well as eccentric muscle actions during resistance exercise have been reported to increase total and free testosterone levels in males (16). Heavy resistance exercise has been shown to significantly increase circulating levels of total and free testosterone in younger and older men, with greater responses found in younger than older men (178). These studies reveal the positive effects of acute and chronic exercise on increased circulating testosterone levels.

As a steroid hormone, circulating testosterone can permeate the blood-brain barrier (179) to interact with androgen receptors, which are located in multiple regions of the brain (180), including the human hippocampus (181). Additionally, testosterone in the hippocampus can be converted by 5α-reductase to DHT, which has a strong binding affinity for androgen receptors in the brain (169). There is some evidence that androgen signaling increases adult neurogenesis in the dentate gyrus of the hippocampus. For example, two weeks of light exercise has been shown to increase synthesis of 5α-reductase, DHT, and androgen receptors in the hippocampus of adult rats, and pharmacological antagonism of androgen receptors blocked exercise-induced neurogenesis (182). Additionally, systemic administration of testosterone or DHT, but not estradiol, was found to enhance hippocampal neurogenesis in male rats (183). However, in a separate study, DHT administration had inconsistent effects on hippocampal proliferation and the number of newborn hippocampal neurons in rats of different age and sex, thereby indicating that the pro-neurogenic effects of androgens on neurogenesis are sex- and age-dependent (184). Interestingly, the manner in which the gonadal steroids, testosterone and estrogen, act on neurogenesis differs in that estrogens appear to induce cell proliferation, whereas androgens increase neuron number via increasing cell survival (185). The pro-survival effects of androgen signaling on hippocampal neurons has been suggested to involve upregulation of BDNF and PKC-dependent phosphorylation of CREB, among other pathways (169). Further investigations are needed to understand the degree to which androgen-stimulated neurogenesis influences cognitive performance in individuals of different age and sex, as well as how such effects are modulated by different forms of exercise. However, a variety of studies have reported positive effects of androgen signaling on spatial working memory (186, 187), and some of the memory-enhancing effects have been verified using memory tasks affected by neurogenesis (169). Considering that andropause, a decline in circulating andogens, occurs in males with aging (188), utilizing exercise to increase circulating levels could represent an important method for maintaining cognitive function.

## Dehydroepiandrosterone

Dehydroepiandrosterone is an endogenous steroid hormone produced in the adrenal glands and nervous system from pregnenolone in the delta-5 pathway via activity of the enzyme cytochrome P450c17 in humans (189). Circulating DHEA can be converted to androstenedione or androstenediol, which subsequently can be converted to testosterone (190). Circulating DHEA declines with age and regulates hippocampal neurogenesis as well as regulates suppression effects of cortisol on formation and survival of new neurons (191). DHEA and its sulfated form, DHEAS, bind to GABA receptors and alter neurosecretion affected by N-Methyl-D-Aspartate (NMDA) receptors (192, 193). In addition, DHEA crosses blood-brain barrier (194). A study of post-menopausal women both on and off of estrogen and progestin replacement therapy reported increases in DHEA and its sulfated form, DHEAS, in response to 30 min of high intensity treadmill running (80% of VO2max) (195). Moreover, the DHEA responses to exercise were greater in those taking hormone replacement therapy, suggesting that women with higher circulating estrogen concentrations would have greater DHEA responses to exercise. A study involving teenage female runners over the course of 7 weeks of a competitive season examined the effects of a graded treadmill test to exhaustion on DHEA and DHEAS across the 7 weeks. The investigators reported significant increases in DHEA and DHEAS in response to each of the graded treadmill tests after weeks 1, 4, and 7; However, the increases in DHEA and DHEAS were similar in response to each exercise bout even through participants aerobic fitness level (VO_2max_) increased across the season (177). In an investigation of well-trained and untrained young adults, DHEA concentrations increased in the untrained in response to 15-min bouts of cycle ergometry at 40% and 70% VO_2_peak, and increased cycling at 100% of VO_2_peak until exhaustion; however, DHEA only increased in the well-trained young adults after exercising at 100% VO_2_peak (196). Results altogether suggest that well trained individuals would need to exercise at higher intensities to stimulate increases in circulating DHEA. A recent systematic review of exercise training studies found that regular training of both males and females over the age of 40 increased circulating basal levels of DHEA as well as testosterone and GH (114). In the harvested rat brain cortex, DHEA treatment increased neurotrophin expression as well as neurite extension (197). An older study revealed that subcutaneaous treatment of male rats with DHEA pellets caused increases in newly formed cells in the dentate gyrus of the hippocampus (191). It also reduced the suppressive effect of corticosterone. A review of studies investigating the effectiveness of DHEA for treatment of older adults for dementia reported that there was not enough positive evidence for use of DHEA to treat dementia (193).

## Irisin

In 2010, Bostrom et al. discovered a peroxisome proliferator-activated receptor-gamma co-activator 1α (PGC-1α)-dependent myokine named irisin and demonstrated that PGC-1α stimulates expression of the membrane protein FNDC5, which is cleaved and released from muscle as the hormone irisin (198). The irisin receptors in the brain were recently identified as integrin αVβ5 heterodimers (199).

A skeletal muscle response to exercise is the expression of the transcriptional co-activator, PGC-1α (200). Fittingly, numerous studies have revealed increases in irisin in response to different forms of exercise, including moderate to high intensity exercise (201). The first author has reported increases in circulating irisin in men and women in response to 90 min of moderate (60% VO_2_max) treadmill exercise (18). Qui et al. (202) also reported increases in irisin in response to aerobic exercise (cycling and running). Interestingly, irisin is significantly lower in patients with Alzheimer’s disease (203). However, there is recent evidence that resistance exercise training increases circulating irisin levels in older men. Zhao et al. (204) compared circulating irisin concentrations in older men who had performed resistance training 2x/wk for 12 weeks versus a control group. They reported that the trained group had significantly higher irisin resting levels than the group that did not perform resistance training. Interestingly, the effect of exercise training on irisin levels is influenced by environmental temperature. McCormick et al. (205) recently conducted a study comparing irisin responses to exercise in older and younger men under hotter and more temperate conditions. They reported elevated irisin responses to aerobic exercise in hotter conditions in younger and older men with greater responses in younger men. Jurimae et al. (206) recently reported that three weeks of sprint interval training in older men (63+/-8 y) significantly increased resting circulating irisin concentrations while reducing inflammatory cytokines.

Both FNDC5 and irisin have been found in mouse and human brains (21, 207), and exercise has been shown to increase hippocampal FNDC5 levels and upregulate the expression of BDNF. Additionally, knockdown of FNDC5 has been reported to reduce central BDNF expression (208), while adenoviral mediated irisin expression increases BDNF in hippocampal cultures (207, 209). Cyclic AMP element response binding protein (CREB) is a cellular transcription factor known for inducing neuronal plasticity and long-term memory formation in the brain (210). Lourenco et al. found that in human cortical slices, recombinant irisin stimulated the cAMP/PKA/CREB pathway (21). When considered with the aforementioned evidence for the role of BDNF and CREB signaling in the regulation of hippocampal neurogenesis, these findings suggest that beneficial effects of different forms of exercise in younger and older individuals may occur through positive effects on neurogenesis mediated by increased circulating irisin concentrations.

## Vascular endothelial growth factor

Vascular endothelial growth factor (VEGF) is an important angiogenic factor for endothelial cells. Platelets are known to be major contributors of circulating VEGF (211). VEGF binds to two tyrosine kinase receptors (VEGFRs), VEGFR-1 and VEGFR-2 (212).

Multiple forms of exercise have been reported to stimulate increases in VEGF. For example, acute sprint training has been shown to increase circulating levels of VEGF (213). A study of older men (72 +/- 6.5y) who performed 5 sets of unilateral leg extensions at 20% 1-repetition maximum of both limbs using either vascular occlusion or no vascular occlusion, reported increased circulating concentrations of VEGF as well as GH in response to the resistance exercise with vascular occlusion (214). A recent investigation on effects of only 15 min of aerobic exercise on circulating VEGF, GH and IGF-1 reported no change in older men and women, suggesting longer bouts of aerobic exercise may be required for alterations in these hormones (215). They also reported that cerebral blood flow increased in the hippocampus of the participants including those with genetic risk factor for Alzheimer’s. A recent study compared the effects of cycling at 60% of VO_2_max in older and younger participants on VEGF and reported significant increases immediately following exercise in older, but not younger participants (216). Three hours post-exercise VEGF values were at baseline levels. Another recent study compared the effects of training that included walking and resistance band exercise 3x/week for 12 weeks in young/old (65-74y) and old/old (75-84y) individuals (217). They found increases in resting VEGF levels in the young/old but not the old/old group after 12 weeks of training, suggesting that this form of training in individuals >74y may not increase resting VEGF values.

A large number of studies have shown that VEGF receptors VEGFR-1 and VEGFR-2 are expressed in neurons (218). VEGF can exert a variety of important functions in the brain, including increasing the permeability of the blood-brain barrier (219) and regulating blood flow in the hippocampus to stimulate neurogenesis (220). A recent review of studies determining the effects of acute aerobic and resistance exercise on VEGF as well as BDNF reported that both forms of exercise increase VEGF and BDNF to potentially affect neurogenesis (221). VEGF-R1 is prevalent in postnatal neurons of the cortex, striatum, and hippocampus but declines with age; however, VEGF-R2 signaling has been shown to lead to proliferation, migration and differentiation of neurons, with expression persisting during adulthood. (218). Sun et al. (222), using a 3-day old rat model, reported increased angiogenesis via altering VEGF with concomitant neural stem cell proliferation and differentiation in the premature brain. Another study found that implantation of biodegradable nanospheres of VEGF in the cerebral cortex of a transgenic mouse model of Alzheimer’s Disease caused cellular proliferation in the hippocampus and dentate gyrus (223). Thus, evidence indicates that VEGF responses to exercise could play a positive role in neurogenesis.

## Erythropoeitin

Erythropoeitin (EPO) is produced by the peritubular cells of the cortex/medullary border of the kidney (224). EPO gene expression is stimulated by hypoxia that will induce elevated red blood cell number, hemoglobin levels and O_2_ capacity in the blood (225). Binding of EPO to the EPO-R results in receptor trans-autophosphorylation, resulting in activation of JAK2-STAT5, PI3-kinase, PKC, and MAPK pathways (226, 227).

EPO levels have been shown to increase in women completing three sets of 12 repetitions of bench press, dumbbell curl, dumbbell squat, and standing dumbbell upright row at either 60%, 70%, or 80% of one-repetition maximum. EPO levels increased to the greatest degree in the groups completing the exercise at 80% one-repetition maximum (228), revealing that heavier workloads resulted in greater EPO response. Female runners completing a marathon were found to not have increased erythropoeitin levels until three days following the run (229). After running a marathon, EPO levels have been shown to increase in male runners; however, the increases were reported to be dependent upon serum iron levels (230). Another study reported a 26% increase in EPO levels in eight males following completion of a half-marathon (231). A recent study compared EPO responses of participants (age 31+/- 6y) to running at high intensity for 30 min vs. running at moderate intensity for 90 min. The moderate intensity runners showed increases in EPO during exercise that returned to baseline at the end of the exercise bout, but there was no significant change in EPO in the high intensity runners (232). Another investigation examined the effects of eight weeks of 1-hr cycle ergometry training sessions, three to four times per week for eight weeks, on regulators of erythropoiesis (233). They reported mild, transient increases in EPO with training over the 8 weeks. A recent study compared circulating EPO levels in men cycling at 60% of power output at VO_2_max in an environmental chamber under either hot-hypoxic, hypoxic, or normoxic conditions (234). They reported increases in EPO after the hot-hypoxic and hypoxic conditions, but not under normoxic conditions. Altogether, results of these studies suggest there are increases in EPO in response to fairly intense resistance exercise and long aerobic exercise bouts. There is some evidence that longer, less intense aerobic exercise results in EPO increases; whereas shorter, more intense aerobic exercise does not. Aerobic training appears to cause transient increases in resting EPO. Finally, moderate exercise under hot or high altitude conditions appear to increase circulating EPO concentrations.

Despite its large molecular weight and susceptibility to glycosylation, circulating EPO is able to cross the blood-brain barrier (235, 236). EPO-R brain expression has been observed during development and adulthood in humans, non-human primates, and other mammals; and the binding of I^125^–labeled EPO localized EPO binding sites in the hippocampus, cortex and midbrain in mouse. While brain expression of EPO-R is low during adulthood, expression of the receptor increases in response to hypoxia or other types of stress (237). During the past two decades, an abundance of studies has established that circulating EPO can exert robust neuroprotective effects in the brain. For example, systemic administration of EPO has been found to reduce neural tissue damage in mouse models of ischemia, traumatic brain injury, autoimmune encephalitis, seizures, Alzheimer’s disease, and amyotrophic lateral sclerosis (227, 236, 238–240). The beneficial properties of EPO in the brain likely relate to the ability of EPO-R signaling to stimulate anti-apoptotic proteins such as B-cell lymphoma 2 (Bcl2) and B-cell lymphoma-extra large (BclxL) (241), to inhibit pro-apoptotic proteins cytochrome-c and p53 (242, 243), and to promote the release of anti-inflammatory cytokines (239, 227). However, such beneficial effects may also relate to modulation of neurogenesis. For example, in a rat model of Alzheimer’s disease, systemic injection of EPO enhanced neuronal proliferation in the dentate gyrus (244). Additionally, a recent study by Wakhloo and colleagues indicates that cognitive challenge induces local hypoxia in hippocampal pyramidal neurons, thereby stimulating upregulation of EPO and EPO-R. Subsequently, EPO signaling promotes the formation of new hippocampal pyramidal neurons and enhances dendritic spine densities (245). Given this evidence for the role of EPO signaling in facilitating hippocampal circuitry formation in response to cognitively-demanding tasks, and considering the aforementioned effects of exercise in increasing circulating EPO, EPO may serve as a central mediator of exercise-induced cognitive benefits.

## Cortisol

Cortisol is a glucocorticoid (GC) hormone that is released from the adrenal glands in response to activation of the hypothalamic-pituitary-adrenal (HPA) axis (246). Higher levels of stress will stimulate the neurons in the paraventricular nucleus of the hypothalamus to secrete corticotropin-releasing hormone (CRH) into the hypophyseal portal system, thereby stimulating the anterior pituitary to release adrenocorticotropic hormone (ACTH) (246). ACTH will circulate to the adrenal glands and stimulate the release of cortisol into circulation. Cortisol, a glucocorticoid hormone, will bind to corticosteroid binding globulin and be carried in the blood (247). Corticosteroid-binding globulin (CBG) is the main GC-binding protein in the plasma, with about 80–90% of the GCs bound to it. It will circulate in the blood stream and bind to mineral corticoid receptors and glucocorticoid receptors (248). Cortisol and other glucocorticoids are soluble lipids that easily cross the blood-brain barrier and are able to bind to glucocorticoid receptors in the amygdala, prefrontal cortex, and the hippocampus (249).

Exercise at higher intensities as well as extended exercise duration at moderate intensities will activate the HPA axis (250, 251). For example, a study involving adolescent female runners examined hormone responses to a maximal graded exercise test at week 1, 4, and 7 of a high school track season. There were significant increases in circulating cortisol levels in response to each of the graded exercise tests to max. However, there were no changes in resting cortisol levels over the 7-week time period (177). A separate study compared the effects of 30 minutes of treadmill exercise at 80% of VO_2_max in postmenopausal women on and off of hormone replacement therapy (HRT) (250). Results revealed that the strenuous exercise increased cortisol levels in both groups, but women on HRT had significantly higher cortisol responses than those not on HRT (195).

In addition to exercise at higher intensities, mental stress with lower exercise intensities has been shown to increase circulating cortisol levels (250). However, lower intensity exercise without mental stress has been shown to not increase circulating cortisol levels (250, 252). Similarly, treadmill running by male and female 10K runners for 30 min at 80% heart rate maximum was shown to not increase circulating cortisol levels (20). A study on effects of 3 sets of 4 resistance exercises (bench press, lat-pull, leg extension, and leg curl) at a 10-repetition maximum load (a moderate intensity), revealed no change in circulating cortisol concentrations in young men (253). Another study was designed to compare the effect of acute psychological stress and moderate as well as vigorous exercise on intense HPA responses and working memory performance. Salivary cortisol concentrations were increased similarly by vigorous exercise and by psychological stress, but not by moderate exercise (254).

The effects of exercise training on circulating cortisol were analyzed in a recent systematic review (114). The results suggested there was not a consensus on whether exercise training changed resting circulating cortisol levels. Another recent study examined the effects of training on resting cortisol levels. The investigators examined the effects of six types of training for six weeks on resting cortisol levels. The training groups were endurance running, endurance/interval running, resistance training, explosive training, speed-endurance 50-meter running, and speed-endurance training. Results from the study revealed that the endurance training groups and strength training programs reduced resting cortisol levels (255).

In the brain, cortisol can bind to receptors and subsequently inhibit expression of a variety of specific genes (256). It has been shown that activation of the glucocorticoid receptor (GR) results in an increase in the expression of serum- and glucocorticoid-inducible kinase 1 (SGK1) in human stem cells and neurons of rodents (257, 258) and that SGK1 mediates a cortisol-induced reduction in neurogenesis (259). Furthermore, a separate study revealed that GC signaling promotes apoptosis in NPCs and immature hippocampal neurons (260). Although there is evidence of a negative effect of cortisol on neurogenesis, it should also be kept in mind that only approximately 10% of circulating cortisol is able to cross the blood-brain barrier (261). Nonetheless, a recent study examined the effects of rigorous resistance exercise in military trained power-lifting subjects on salivary cortisol, memory, and learning ability. After completing a strenuous resistance exercise protocol, the subjects’ salivary cortisol levels had significantly increased and importantly, their learning ability and memory was reduced (249).

Collectively, these findings suggest that low and moderate training intensities will not acutely affect circulating cortisol levels, but high intensity exercise can increase circulating cortisol and potentially result in an inhibitory effect on neurogenesis. However, there is evidence that endurance training and strength training of moderate intensities could reduce resting cortisol levels, thereby conferring a positive effect on neurogenesis.

## Discussion

**Figure 1:**
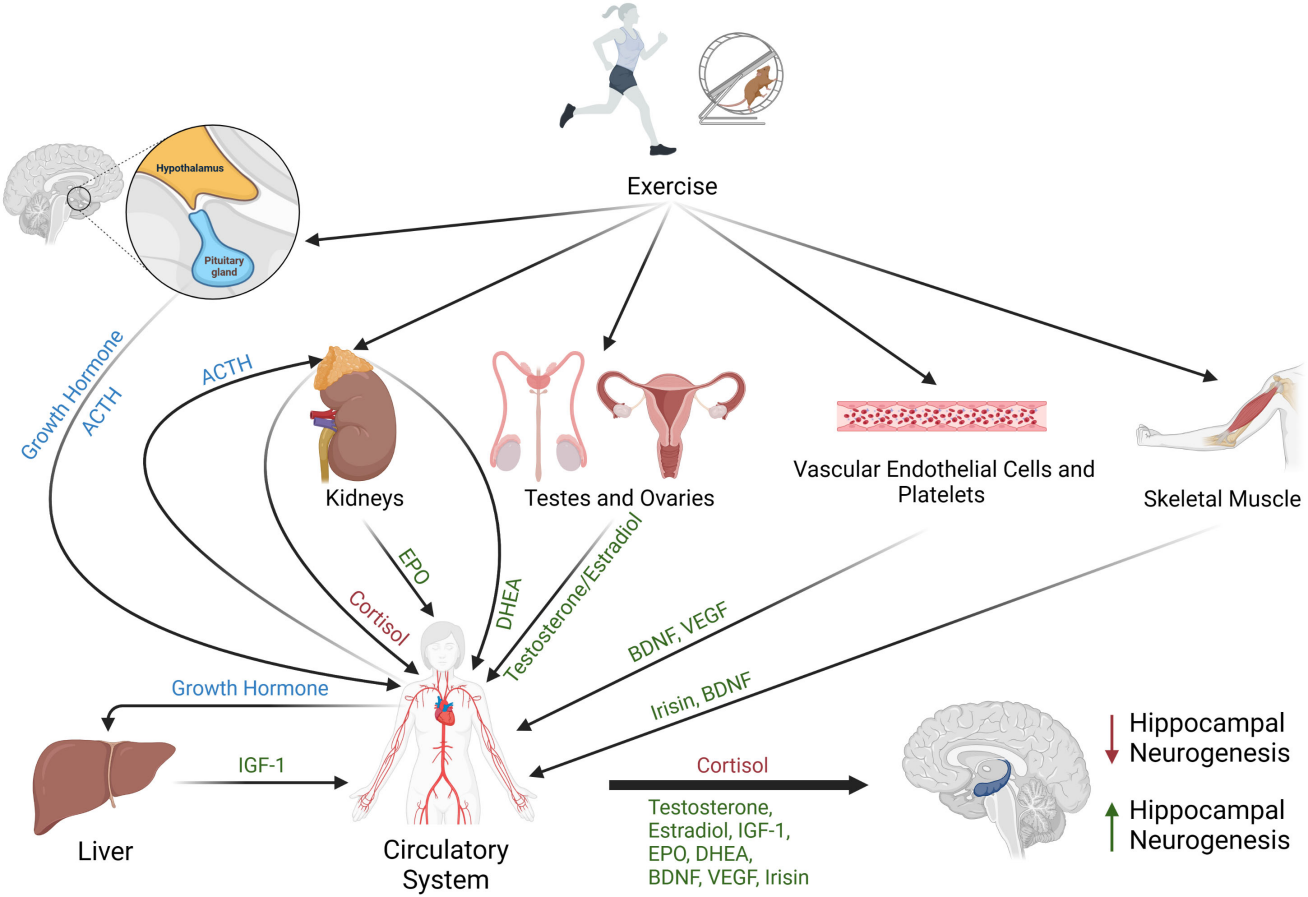
Schematic representing the effects of exercise on circulating hormones that regulate neurogenesis. Different forms of exercise may stimulate the release of brain-derived neurotrophic factor (BDNF) from vascular endothelial cells, platelets, skeletal muscle, or the brain. Exercise may also trigger the secretion of the myokine irisin from skeletal muscle, promote release of testosterone and estradiol from testes or ovaries, stimulate release of vascular endothelial growth factor (VEGF) from platelets, signal adrenal glands to release DHEA and cortisol, stimulate the kidneys to release erythropoietin (EPO), and induce secretion of growth hormone from the pituitary gland. Circulating growth hormone stimulates production and release of insulin-like-growth factor-1 (IGF-1) from the liver. Collectively, increased circulating levels of BDNF, GH, IGF-1, EPO, estrogen, testosterone, DHEA, irisin, and VEGF are associated with increased hippocampal neurogenesis (green), while elevated circulating cortisol is associated with decreased neurogenesis (red).

In summary, different forms of exercise increase circulating levels of a broad range of hormones. Numerous investigations have revealed important roles for these endocrine factors in the stimulation of neurogenesis (see **Figure 1**), and substantial evidence indicates that exercise-induced changes in these factors can positively affect the maintenance and improvement of cognitive function. There can be differences in circulating levels of the aforementioned hormones with variation in exercise mode, duration, and intensity, as well as effects of age, training status, and gender. Future studies are needed to evaluate combinatorial signaling between these endocrine factors, as well as identify the most effective training methods to increase these circulating hormones to promote neurogenesis in patients with traumatic brain injury or neurogenerative disease. These data will be important for recommending exercise regimens that will effectively increase neurogenesis. Additionally, more data may facilitate the development of effective procedures for infusion of hormones to treat patients with neurodegeneration.

## Author contributions

RK and BK contributed to the conception of the review. RK and BK retrieved the main articles to be included in the review. RK and BK wrote the first draft of the manuscript. BK developed the figure. All authors contributed to the article and approved the submitted version.

## References

[1] Braun SMG Jessberger S. Adult neurogenesis: mechanisms and functional significance. Development (2014) 141:1983–6. doi: 10.1242/DEV.104596 24803647

[2] Toda T Parylak SL Linker SB Gage FH. The role of adult hippocampal neurogenesis in brain health and disease. Mol Psychiatry (2018) 24:67–87. doi: 10.1038/s41380-018-0036-2 29679070 PMC6195869

[3] Trinchero MF Herrero M Schinder AF. Rejuvenating the brain with chronic exercise through adult neurogenesis. Front Neurosci (2019) 13:1000. doi: 10.3389/FNINS.2019.01000 31619959 PMC6759473

[4] Bednarczyk MR Aumont A Décary S Bergeron R Fernandes KJL. Prolonged voluntary wheel-running stimulates neural precursors in the hippocampus and forebrain of adult CD1 mice. Hippocampus (2009) 19:913–27. doi: 10.1002/HIPO.20621 19405143

[5] Erickson KI Leckie RL Weinstein AM. Physical activity, fitness, and gray matter volume. Neurobiol Aging (2014) 35 (Suppl 2):S20–S28. doi: 10.1016/J.NEUROBIOLAGING.2014.03.034 PMC4094356 24952993

[6] Cooper C Moon HY Van Praag H. On the run for hippocampal plasticity. Cold Spring Harb Perspect Med (2018) 8(4):a029736. doi: 10.1101/CSHPERSPECT.A029736 28495803 PMC5880155

[7] Ben-Zeev T Shoenfeld Y Hoffman JR. The Effect of Exercise on Neurogenesis in the Brain - PubMed (Accessed April 3, 2023). 35971998

[8] Hötting K Röder B. Beneficial effects of physical exercise on neuroplasticity and cognition. Neurosci Biobehav Rev (2013) 37:2243–57. doi: 10.1016/J.NEUBIOREV.2013.04.005 23623982

[9] Saraulli D Costanzi M Mastrorilli V Farioli-Vecchioli S. The long run: neuroprotective effects of physical exercise on adult neurogenesis from youth to old age. Curr Neuropharmacol (2017) 15:519–33. doi: 10.2174/1570159X14666160412150223 PMC5543673 27000776

[10] Sujkowski A Hong L Wessells RJ Todi SV. The protective role of exercise against age-related neurodegeneration. Ageing Res Rev (2022) 74:101543. doi: 10.1016/J.ARR.2021.101543 34923167 PMC8761166

[11] Deslandes A Moraes H Ferreira C Veiga H Silveira H Mouta R. Exercise and mental health: many reasons to move. Neuropsychobiology (2009) 59:191–8. doi: 10.1159/000223730 19521110

[12] Kraemer RR Kilgore JL Kraemer GR Daniel Castracane V. Growth hormone, igf-i, and testosterone responses to resistive exercise. Med Sci Sports Exerc (1992) 24:1346–52. doi: 10.1249/00005768-199212000-00007 1470017

[13] Kraemer RR Durand RJ Acevedo EO Johnson LG Kraemer GR Hebert EP. Rigorous running increases growth hormone and insulin-like growth factor-I without altering ghrelin. Exp Biol Med (Maywood) (2004) 229:240–6. doi: 10.1177/153537020422900304 14988516

[14] Kraemer RR Francois MR Sehgal K Sirikul B Valverde RA Castracane VD. Amylin and selective glucoregulatory peptide alterations during prolonged exercise. Med Sci Sports Exerc (2011) 43:1451–6. doi: 10.1249/MSS.0B013E3182114AB9 21266924

[15] Acevedo EO Kraemer RR Kamimori GH Durand RJ Johnson LG Castracane VD. Stress hormones, effort sense, and perceptions of stress during incremental exercise: an exploratory investigation. J Strength Cond Res (2007) 21:283–8. doi: 10.1519/00124278-200702000-00050 17313283

[16] Durand RJ Castracane VD Hollander DB Tryniecki JL Bamman MM O’Neal S. Hormonal responses from concentric and eccentric muscle contractions. Med Sci Sports Exerc (2003) 35:937–43. doi: 10.1249/01.MSS.0000069522.38141.0B 12783041

[17] Kraemer RR Heleniak RJ Tryniecki JL Kraemer GR Okazaki NJ Castracane VD. Follicular and luteal phase hormonal responses to low-volume resistive exercise. Med Sci Sports Exerc (1995) 27:809–17. doi: 10.1249/00005768-199506000-00004 7658941

[18] Kraemer RR Shockett P Webb ND Shah U Castracane VD. A transient elevated irisin blood concentration in response to prolonged, moderate aerobic exercise in young men and women. Horm Metab Res (2014) 46:150–4. doi: 10.1055/S-0033-1355381 24062088

[19] Kraemer RR Goldfarb AH Reeves GV Meachum WA Daniel Castracane V. Effects of partial vascular occlusion on irisin responses to loaded muscle contractions. Appl Physiol Nutr Metab (2016) 41:332–4. doi: 10.1139/APNM-2015-0464 26859524

[20] Kraemer RR Blair S Kraemer GR Castracane VD. Effects of treadmill running on plasma beta-endorphin, corticotropin, and cortisol levels in male and female 10K runners. Eur J Appl Physiol Occup Physiol (1989) 58:845–51. doi: 10.1007/BF02332217 2548864

[21] Lourenco MV Frozza RL de Freitas GB Zhang H Kincheski GC Ribeiro FC. Exercise-linked FNDC5/irisin rescues synaptic plasticity and memory defects in Alzheimer’s models. Nat Med (2019) 25:165–75. doi: 10.1038/S41591-018-0275-4 PMC6327967 30617325

[22] Lee C Agoston DV. Vascular endothelial growth factor is involved in mediating increased *de novo* hippocampal neurogenesis in response to traumatic brain injury. J Neurotrauma (2010) 27:541–53. doi: 10.1089/NEU.2009.0905 20001687

[23] Guan J Mathai S Liang HP Gunn AJ. Insulin-like growth factor-1 and its derivatives: potential pharmaceutical application for treating neurological conditions. Recent Pat CNS Drug Discovery (2013) 8:142–60. doi: 10.2174/1574889811308020004 23597305

[24] Kraemer BR Carter BD. Receptors | Neurotrophin Receptor Signaling. In: Encyclopedia of Biological Chemistry, 3rd ed. (Cambridge, MA, United States: Elsevier Inc.), vol. 6. (2021). p. 187–200. doi: 10.1016/B978-0-12-819460-7.00310-8

[25] Numakawa T Yokomaku D Richards M Hori H Adachi N Kunugi H. Functional interactions between steroid hormones and neurotrophin BDNF. World J Biol Chem (2010) 1:133. doi: 10.4331/WJBC.V1.I5.133 21540998 PMC3083963

[26] Kraemer BR Yoon SO Carter BD. The biological functions and signaling mechanisms of the p75 neurotrophin receptor. Handb Exp Pharmacol (2014) 220:121–64. doi: 10.1007/978-3-642-45106-5_6 24668472

[27] Hofer M Pagliusi SR Hohn A Leibrock J Barde YA. Regional distribution of brain-derived neurotrophic factor mRNA in the adult mouse brain. EMBO J (1990) 9:2459–64. doi: 10.1002/J.1460-2075.1990.TB07423.X PMC552273 2369898

[28] Miranda M Morici JF Zanoni MB Bekinschtein P. Brain-derived neurotrophic factor: A key molecule for memory in the healthy and the pathological brain. Front Cell Neurosci (2019) 13:363. doi: 10.3389/FNCEL.2019.00363 31440144 PMC6692714

[29] Helan M Aravamudan B Hartman WR Thompson MA Johnson BD Pabelick CM. BDNF secretion by human pulmonary artery endothelial cells in response to hypoxia. J Mol Cell Cardiol (2014) 68:89–97. doi: 10.1016/J.YJMCC.2014.01.006 24462831 PMC3977651

[30] Prigent-Tessier A Quirie A Maguin-Gate K Szostak J Mossiat C Nappey M. Physical training and hypertension have opposite effects on endothelial brain-derived neurotrophic factor expression. Cardiovasc Res (2013) 100:374–82. doi: 10.1093/CVR/CVT219 24092446

[31] Kerschensteiner M Gallmeier E Behrens L Leal VV Misgeld T Klinkert WEF. Activated human T cells, B cells, and monocytes produce brain-derived neurotrophic factor in *vitro* and in inflammatory brain lesions: a neuroprotective role of inflammation? J Exp Med (1999) 189:865–70. doi: 10.1084/JEM.189.5.865 PMC2192942 10049950

[32] Walsh JJ Edgett BA Tschakovsky ME Gurd BJ. Fasting and exercise differentially regulate BDNF mRNA expression in human skeletal muscle. Appl Physiol Nutr Metab (2015) 40:96–8. doi: 10.1139/APNM-2014-0290 25494871

[33] Matthews VB Åström MB Chan MHS Bruce CR Krabbe KS Prelovsek O. Brain-derived neurotrophic factor is produced by skeletal muscle cells in response to contraction and enhances fat oxidation via activation of AMP-activated protein kinase. Diabetologia (2009) 52:1409–18. doi: 10.1007/S00125-009-1364-1 19387610

[34] Walsh JJ Tschakovsky ME. Exercise and circulating BDNF: Mechanisms of release and implications for the design of exercise interventions. Appl Physiol Nutr Metab (2018) 43:1095–104. doi: 10.1139/APNM-2018-0192 29775542

[35] Zagrebelsky M Korte M. Form follows function: BDNF and its involvement in sculpting the function and structure of synapses. Neuropharmacology (2014) 76 Pt C:628–38. doi: 10.1016/J.NEUROPHARM.2013.05.029 23752094

[36] Yang J Siao CJ Nagappan G Marinic T Jing D McGrath K. Neuronal release of proBDNF. Nat Neurosci (2009) 12:113–5. doi: 10.1038/NN.2244 PMC2737352 19136973

[37] Numakawa T Odaka H Adachi N. Actions of brain-derived neurotrophin factor in the neurogenesis and neuronal function, and its involvement in the pathophysiology of brain diseases. Int J Mol Sci (2018) 19. doi: 10.3390/IJMS19113650 PMC6274766 30463271

[38] Li T Jiang L Zhang X Chen H. *In-vitro* effects of brain-derived neurotrophic factor on neural progenitor/stem cells from rat hippocampus. Neuroreport (2009) 20:295–300. doi: 10.1097/WNR.0B013E32832000C8 19188859

[39] Wei Z Liao J Qi F Meng Z Pan S. Evidence for the contribution of BDNF-TrkB signal strength in neurogenesis: An organotypic study. Neurosci Lett (2015) 606:48–52. doi: 10.1016/J.NEULET.2015.08.032 26306653

[40] Ortiz-López L Vega-Rivera NM Babu H Ramírez-Rodríguez GB. Brain-derived neurotrophic factor induces cell survival and the migration of murine adult hippocampal precursor cells during differentiation *in vitro* . Neurotox Res (2017) 31:122–35. doi: 10.1007/S12640-016-9673-X 27663583

[41] Lee J Duan W Mattson MP. Evidence that brain-derived neurotrophic factor is required for basal neurogenesis and mediates, in part, the enhancement of neurogenesis by dietary restriction in the hippocampus of adult mice. J Neurochem (2002) 82:1367–75. doi: 10.1046/J.1471-4159.2002.01085.X 12354284

[42] Scharfman H Goodman J Macleod A Phani S Antonelli C Croll S. Increased neurogenesis and the ectopic granule cells after intrahippocampal BDNF infusion in adult rats. Exp Neurol (2005) 192:348–56. doi: 10.1016/J.EXPNEUROL.2004.11.016 15755552

[43] Ribeiro FF Xapelli S. Intervention of brain-derived neurotrophic factor and other neurotrophins in adult neurogenesis. Adv Exp Med Biol (2021) 1331:95–115. doi: 10.1007/978-3-030-74046-7_8 34453295

[44] Erickson KI Prakash RS Voss MW Chaddock L Heo S McLaren M. Brain-derived neurotrophic factor is associated with age-related decline in hippocampal volume. J Neurosci (2010) 30:5368–75. doi: 10.1523/JNEUROSCI.6251-09.2010 PMC3069644 20392958

[45] Ziegenhorn AA Schulte-Herbrüggen O Danker-Hopfe H Malbranc M Hartung HD Anders D. Serum neurotrophins–a study on the time course and influencing factors in a large old age sample. Neurobiol Aging (2007) 28:1436–45. doi: 10.1016/J.NEUROBIOLAGING.2006.06.011 16879899

[46] Karege F Schwald M Cisse M. Postnatal developmental profile of brain-derived neurotrophic factor in rat brain and platelets. Neurosci Lett (2002) 328:261–4. doi: 10.1016/S0304-3940(02)00529-3 12147321

[47] Hayashi M Mistunaga F Ohira K Shimizu K. Changes in BDNF-immunoreactive structures in the hippocampal formation of the aged macaque monkey. Brain Res (2001) 918:191–6. doi: 10.1016/S0006-8993(01)03002-5 11684059

[48] Newton IG Forbes ME Legault C Johnson JE Brunso-Bechtold JK Riddle DR. Caloric restriction does not reverse aging-related changes in hippocampal BDNF. Neurobiol Aging (2005) 26:683–8. doi: 10.1016/J.NEUROBIOLAGING.2004.06.005 15708443

[49] Weinstein G Beiser AS Choi SH Preis SR Chen TC Vorgas D. Serum brain-derived neurotrophic factor and the risk for dementia: the Framingham Heart Study. JAMA Neurol (2014) 71:55–61. doi: 10.1001/JAMANEUROL.2013.4781 24276217 PMC4056186

[50] Mizoguchi Y Yao H Imamura Y Hashimoto M Monji A. Lower brain-derived neurotrophic factor levels are associated with age-related memory impairment in community-dwelling older adults: the Sefuri study. Sci Rep (2020) 10:1–9. doi: 10.1038/s41598-020-73576-1 33020545 PMC7536184

[51] Nagahara AH Merrill DA Coppola G Tsukada S Schroeder BE Shaked GM. Neuroprotective effects of brain-derived neurotrophic factor in rodent and primate models of Alzheimer’s disease. Nat Med (2009) 15:331. doi: 10.1038/NM.1912 19198615 PMC2838375

[52] Connor B Sun Y Von Hieber D Tang SK Jones KS Maucksch C. AAV1/2-mediated BDNF gene therapy in a transgenic rat model of Huntington’s disease. Gene Ther (2016) 23:283–95. doi: 10.1038/GT.2015.113 26704721

[53] Real CC Ferreira AFB Chaves-Kirsten GP Torrão AS Pires RS Britto LRG. BDNF receptor blockade hinders the beneficial effects of exercise in a rat model of Parkinson’s disease. Neuroscience (2013) 237:118–29. doi: 10.1016/J.NEUROSCIENCE.2013.01.060 23396085

[54] Arazi H Babaei P Moghimi M Asadi A. Acute effects of strength and endurance exercise on serum BDNF and IGF-1 levels in older men. BMC Geriatr (2021) 21:50. doi: 10.1186/S12877-020-01937-6 33441099 PMC7807435

[55] Coelho FG de M Gobbi S Andreatto CAA Corazza DI Pedroso RV Santos-Galduróz RF. Physical exercise modulates peripheral levels of brain-derived neurotrophic factor (BDNF): a systematic review of experimental studies in the elderly. Arch Gerontol Geriatr (2013) 56:10–5. doi: 10.1016/J.ARCHGER.2012.06.003 22749404

[56] Marston KJ Newton MJ Brown BM Rainey-Smith SR Bird S Martins RN. Intense resistance exercise increases peripheral brain-derived neurotrophic factor. J Sci Med Sport (2017) 20:899–903. doi: 10.1016/J.JSAMS.2017.03.015 28511848

[57] Tsai CL Pan CY Tseng YT Chen FC Chang YC Wang TC. Acute effects of high-intensity interval training and moderate-intensity continuous exercise on BDNF and irisin levels and neurocognitive performance in late middle-aged and older adults. Behav Brain Res (2021) 413:113472. doi: 10.1016/J.BBR.2021.113472 34274372

[58] Marquez CMS Vanaudenaerde B Troosters T Wenderoth N. High-intensity interval training evokes larger serum BDNF levels compared with intense continuous exercise. J Appl Physiol (2015) 119:1363–73. doi: 10.1152/JAPPLPHYSIOL.00126.2015/ASSET/IMAGES/LARGE/ZDG0231516450007.JPEG 26472862

[59] El Hayek L Khalifeh M Zibara V Abi Assaad R Emmanuel N Karnib N. Lactate mediates the effects of exercise on learning and memory through SIRT1-dependent activation of hippocampal brain-derived neurotrophic factor (BDNF). J Neurosci (2019) 39:2369–82. doi: 10.1523/JNEUROSCI.1661-18.2019 PMC6435829 30692222

[60] Bonanni R Cariati I Tarantino U D’arcangelo G Tancredi V. Physical exercise and health: A focus on its protective role in neurodegenerative diseases. J Funct Morphol Kinesiol (2022) 7(2):38. doi: 10.3390/JFMK7020038 35645300 PMC9149968

[61] Sleiman SF Henry J Al-Haddad R El Hayek L Haidar EA Stringer T. Exercise promotes the expression of brain derived neurotrophic factor (BDNF) through the action of the ketone body β-hydroxybutyrate. Elife (2016) 5:e15092. doi: 10.7554/ELIFE.15092 27253067 PMC4915811

[62] Ding Q Ying Z Gómez-Pinilla F. Exercise influences hippocampal plasticity by modulating brain-derived neurotrophic factor processing. Neuroscience (2011) 192:773–80. doi: 10.1016/J.NEUROSCIENCE.2011.06.032 PMC3225196 21756980

[63] Ferris LT Williams JS Shen CL. The effect of acute exercise on serum brain-derived neurotrophic factor levels and cognitive function. Med Sci Sports Exerc (2007) 39:728–34. doi: 10.1249/MSS.0B013E31802F04C7 17414812

[64] Nofuji Y Suwa M Sasaki H Ichimiya A Nishichi R Kumagai S. Different circulating brain-derived neurotrophic factor responses to acute exercise between physically active and sedentary subjects. J Sports Sci Med (2012) 11:83. 24137066 PMC3737858

[65] Reycraft JT Islam H Townsend LK Hayward GC Hazell TOMJ MacPherson REK. Exercise intensity and recovery on circulating brain-derived neurotrophic factor. Med Sci Sports Exerc (2020) 52:1210–7. doi: 10.1249/MSS.0000000000002242 31815833

[66] Stimpson NJ Davison G Javadi AH. Joggin’ the noggin: towards a physiological understanding of exercise-induced cognitive benefits. Neurosci Biobehav Rev (2018) 88:177–86. doi: 10.1016/J.NEUBIOREV.2018.03.018 29572187

[67] Pardridge WM Kang YS Buciak JL. Transport of human recombinant brain-derived neurotrophic factor (BDNF) through the rat blood-brain barrier in *vivo* using vector-mediated peptide drug delivery. Pharm Res (1994) 11:738–46. doi: 10.1023/A:1018940732550 8058646

[68] Fujisawa M Takeshita Y Fujikawa S Matsuo K Okamoto M Tamada M. Exploring lipophilic compounds that induce BDNF secretion in astrocytes beyond the BBB using a new multi-cultured human in *vitro* BBB model. J Neuroimmunol (2022) 362:577783. doi: 10.1016/J.JNEUROIM.2021.577783 34902709

[69] Pan W Banks WA Fasold MB Bluth J Kastin AJ. Transport of brain-derived neurotrophic factor across the blood-brain barrier. Neuropharmacology (1998) 37:1553–61. doi: 10.1016/S0028-3908(98)00141-5 9886678

[70] Klein AB Williamson R Santini MA Clemmensen C Ettrup A Rios M. Blood BDNF concentrations reflect brain-tissue BDNF levels across species. Int J Neuropsychopharmacol (2011) 14:347–53. doi: 10.1017/S1461145710000738 20604989

[71] Sartorius A Hellweg R Litzke J Vogt M Dormann C Vollmayr B. Correlations and discrepancies between serum and brain tissue levels of neurotrophins after electroconvulsive treatment in rats. Pharmacopsychiatry (2009) 42:270–6. doi: 10.1055/S-0029-1224162 19924587

[72] Rasmussen P Brassard P Adser H Pedersen MV Leick L Hart E. Evidence for a release of brain-derived neurotrophic factor from the brain during exercise. Exp Physiol (2009) 94:1062–9. doi: 10.1113/EXPPHYSIOL.2009.048512 19666694

[73] Schmidt HD Duman RS. Peripheral BDNF produces antidepressant-like effects in cellular and behavioral models. Neuropsychopharmacology (2010) 35:2378–91. doi: 10.1038/npp.2010.114 PMC2955759 20686454

[74] Vaynman S Ying Z Gomez-Pinilla F. Hippocampal BDNF mediates the efficacy of exercise on synaptic plasticity and cognition. Eur J Neurosci (2004) 20:2580–90. doi: 10.1111/J.1460-9568.2004.03720.X 15548201

[75] Intlekofer KA Berchtold NC Malvaez M Carlos AJ McQuown SC Cunningham MJ. Exercise and sodium butyrate transform a subthreshold learning event into long-term memory via a brain-derived neurotrophic factor-dependent mechanism. Neuropsychopharmacology (2013) 38:2027–34. doi: 10.1038/NPP.2013.104 PMC3746687 23615664

[76] Ferreira FF Ribeiro FF Rodrigues RS Sebastião AM Xapelli S. Brain-derived neurotrophic factor (BDNF) role in cannabinoid-mediated neurogenesis. Front Cell Neurosci (2018) 12:441. doi: 10.3389/FNCEL.2018.00441 30546297 PMC6279918

[77] Li Y Luikart BW Birnbaum S Chen J Kwon CH Kernie SG. TrkB regulates hippocampal neurogenesis and governs sensitivity to antidepressive treatment. Neuron (2008) 59:399–412. doi: 10.1016/J.NEURON.2008.06.023 18701066 PMC2655199

[78] Ma Z Zang T Birnbaum SG Wang Z Johnson JE Zhang CL. TrkB dependent adult hippocampal progenitor differentiation mediates sustained ketamine antidepressant response. Nat Commun (2017) 8(1):1668. doi: 10.1038/S41467-017-01709-8 29162814 PMC5698402

[79] Nakagawa S Kim JE Lee R Malberg JE Chen J Steffen C. Regulation of neurogenesis in adult mouse hippocampus by cAMP and the cAMP response element-binding protein. J Neurosci (2002) 22:3673–82. doi: 10.1523/JNEUROSCI.22-09-03673.2002 PMC6758358 11978843

[80] Zheng X Chen L Chen T Cao M Zhang B Yuan C. The mechanisms of BDNF promoting the proliferation of porcine follicular granulosa cells: role of miR-127 and involvement of the MAPK-ERK1/2 pathway. Anim (Basel) (2023) 13(6):1115. doi: 10.3390/ANI13061115 PMC10044701 36978655

[81] Chae HD Mitton B Lacayo NJ Sakamoto KM. Replication factor C3 is a CREB target gene that regulates cell cycle progression through the modulation of chromatin loading of PCNA. Leukemia (2015) 29:1379–89. doi: 10.1038/LEU.2014.350 PMC4456282 25541153

[82] Impey S McCorkle SR Cha-Molstad H Dwyer JM Yochum GS Boss JM. Defining the CREB regulon: A genome-wide analysis of transcription factor regulatory regions. Cell (2004) 119:1041–54. doi: 10.1016/J.CELL.2004.10.032 15620361

[83] Lee BH Mathews MB. Transcriptional coactivator cAMP response element binding protein mediates induction of the human proliferating cell nuclear antigen promoter by the adenovirus E1A oncoprotein. Proc Natl Acad Sci U.S.A. (1997) 94:4481–6. doi: 10.1073/PNAS.94.9.4481 PMC20748 9114015

[84] Cao M Niu Q Xiang X Yuan C Iqbal T Huang Y. Brain-derived neurotrophic factor regulates ishikawa cell proliferation through the trkB-ERK1/2 signaling pathway. Biomolecules (2020) 10:1645. doi: 10.3390/BIOM10121645 33302387 PMC7762527

[85] Hernandez JM Floyd DH Weilbaecher KN Green PL Boris-Lawrie K. Multiple facets of junD gene expression are atypical among AP-1 family members. Oncogene (2008) 27:4757. doi: 10.1038/ONC.2008.120 18427548 PMC2726657

[86] Sairanen M Lucas G Ernfors P Castrén M Castrén E. Brain-derived neurotrophic factor and antidepressant drugs have different but coordinated effects on neuronal turnover, proliferation, and survival in the adult dentate gyrus. J Neurosci (2005) 25:1089–94. doi: 10.1523/JNEUROSCI.3741-04.2005 PMC6725966 15689544

[87] Choi SH Li Y Parada LF Sisodia SS. Regulation of hippocampal progenitor cell survival, proliferation and dendritic development by BDNF. Mol Neurodegener (2009) 4:1–12. doi: 10.1186/1750-1326-4-52/FIGURES/5 20025751 PMC2806355

[88] Chan JP Cordeira J Calderon GA Iyer LK Rios M. Depletion of central BDNF in mice impedes terminal differentiation of new granule neurons in the adult hippocampus. Mol Cell Neurosci (2008) 39:372–83. doi: 10.1016/J.MCN.2008.07.017 PMC2652348 18718867

[89] Jagasia R Steib K Englberger E Herold S Faus-Kessler T Saxe M. GABA-cAMP response element-binding protein signaling regulates maturation and survival of newly generated neurons in the adult hippocampus. J Neurosci (2009) 29:7966–77. doi: 10.1523/JNEUROSCI.1054-09.2009 PMC2776747 19553437

[90] Groves N O’Keeffe I Lee W Toft A Blackmore D Bandhavkar S. Blockade of TrkB but not p75NTR activates a subpopulation of quiescent neural precursor cells and enhances neurogenesis in the adult mouse hippocampus. Dev Neurobiol (2019) 79:868–79. doi: 10.1002/DNEU.22729 31886631

[91] Tecuatl C Herrrera-López G Martín-Ávila A Yin B Weber S Barrionuevo G. TrkB-mediated activation of the phosphatidylinositol-3-kinase/Akt cascade reduces the damage inflicted by oxygen-glucose deprivation in area CA3 of the rat hippocampus. Eur J Neurosci (2018) 47:1096. doi: 10.1111/EJN.13880 29480936 PMC5938095

[92] Qi D Ouyang C Wang Y Zhang S Ma X Song YJ. HO-1 attenuates hippocampal neurons injury via the activation of BDNF–TrkB–PI3K/Akt signaling pathway in stroke. Brain Res (2014) 1577:69–76. doi: 10.1016/J.BRAINRES.2014.06.031 24997248

[93] Bernabeu RO Longo FM. The p75 neurotrophin receptor is expressed by adult mouse dentate progenitor cells and regulates neuronal and non-neuronal cell genesis. BMC Neurosci (2010) 11:136. doi: 10.1186/1471-2202-11-136 20961458 PMC2987811

[94] Colditz MJ Catts VS Al-Menhali N Osborne GW Bartlett PF Coulson EJ. P75 neurotrophin receptor regulates basal and fluoxetine-stimulated hippocampal neurogenesis. Exp Brain Res (2010) 200:161–7. doi: 10.1007/s00221-009-1947-6 19621217

[95] Catts VS Al-Menhali N Burne THJ Colditz MJ Coulson EJ. The p75 neurotrophin receptor regulates hippocampal neurogenesis and related behaviours. Eur J Neurosci (2008) 28:883–92. doi: 10.1111/j.1460-9568.2008.06390.x 18717734

[96] Zanin JP Montroull LE Volosin M Friedman WJ. The p75 neurotrophin receptor facilitates trkB signaling and function in rat hippocampal neurons. Front Cell Neurosci (2019) 13:485. doi: 10.3389/fncel.2019.00485 31736712 PMC6828739

[97] Chen J Li CR Yang H Liu J Zhang T Jiao SS. proBDNF attenuates hippocampal neurogenesis and induces learning and memory deficits in aged mice. Neurotox Res (2016) 29:47–53. doi: 10.1007/S12640-015-9568-2/FIGURES/3 26459304

[98] Li JY Liu J Manaph NPA Bobrovskaya L Zhou XF. ProBDNF inhibits proliferation, migration and differentiation of mouse neural stem cells. Brain Res (2017) 1668:46–55. doi: 10.1016/J.BRAINRES.2017.05.013 28528122

[99] Sartori CR Vieira AS Ferrari EM Langone F Tongiorgi E Parada CA. The antidepressive effect of the physical exercise correlates with increased levels of mature BDNF, and proBDNF proteolytic cleavage-related genes, p11 and tPA. Neuroscience (2011) 180:9–18. doi: 10.1016/J.NEUROSCIENCE.2011.02.055 21371535

[100] Inoue DS Monteiro PA Gerosa-Neto J Santana PR Peres FP Edwards KM. Acute increases in brain-derived neurotrophic factor following high or moderate-intensity exercise is accompanied with better cognition performance in obese adults. Sci Rep (2020) 10:13493. doi: 10.1038/S41598-020-70326-1 32778721 PMC7417991

[101] Luo L Li C Deng Y Wang Y Meng P Wang Q. High-intensity interval training on neuroplasticity, balance between brain-derived neurotrophic factor and precursor brain-derived neurotrophic factor in poststroke depression rats. J Stroke Cerebrovasc Dis (2019) 28:672–82. doi: 10.1016/J.JSTROKECEREBROVASDIS.2018.11.009 30503681

[102] Park JK Hong YP Lee SJ. Effects of exercise on mature or precursor brain-derived neurotrophic factor pathways in ovariectomized rats. Mol Med Rep (2017) 16:435–40. doi: 10.3892/MMR.2017.6614/HTML 28534952

[103] Kato Y Murakami Y Sohmiya M Nishiki M. Regulation of human growth hormone secretion and its disorders. Intern Med (2002) 41:7–13. doi: 10.2169/INTERNALMEDICINE.41.7 11838603

[104] Dehkhoda F Lee CMM Medina J Brooks AJ. The growth hormone receptor: mechanism of receptor activation, cell signaling, and physiological aspects. Front Endocrinol (Lausanne) (2018) 9:35. doi: 10.3389/FENDO.2018.00035 29487568 PMC5816795

[105] Jamil Sami A. Structure-function relation of somatotropin with reference to molecular modeling. Curr Protein Pept Sci (2007) 8:283–92. doi: 10.2174/138920307780831820 17584122

[106] Bailes J Soloviev M. Insulin-like growth factor-1 (IGF-1) and its monitoring in medical diagnostic and in sports. Biomolecules (2021) 11:1–15. doi: 10.3390/BIOM11020217 PMC7913862 33557137

[107] Laron Z. Insulin-like growth factor 1 (IGF-1): a growth hormone. Mol Pathol (2001) 54:311–6. doi: 10.1136/MP.54.5.311 PMC1187088 11577173

[108] Wilkinson-Berka J Wraight C Werther G. The role of growth hormone, insulin-like growth factor and somatostatin in diabetic retinopathy. Curr Med Chem (2006) 13:3307–17. doi: 10.2174/092986706778773086 17168853

[109] Carro E Nuñez A Busiguina S Torres-Aleman I. Circulating insulin-like growth factor I mediates effects of exercise on the brain. J Neurosci (2000) 20:2926–33. doi: 10.1523/JNEUROSCI.20-08-02926.2000 PMC6772191 10751445

[110] Kraemer RR Hollander DB Reeves GV Francois M Ramadan ZG Meeker B. Similar hormonal responses to concentric and eccentric muscle actions using relative loading. Eur J Appl Physiol (2006) 96:551–7. doi: 10.1007/S00421-005-0094-4 16369814

[111] Kraemer WJ Ratamess NA Hymer WC Nindl BC Fragala MS. Growth hormone(s), testosterone, insulin-like growth factors, and cortisol: roles and integration for cellular development and growth with exercise. Front Endocrinol (Lausanne) (2020) 11:33. doi: 10.3389/FENDO.2020.00033 32158429 PMC7052063

[112] Eliakim A Nemet D Most G Rakover N Pantanowitz M Meckel Y. Effect of gender on the GH-IGF-I response to anaerobic exercise in young adults. J Strength Cond Res (2014) 28:3411–5. doi: 10.1519/JSC.0000000000000605 24983853

[113] Bunt JC Boileau RA Bahr JM Nelson RA. Sex and training differences in human growth hormone levels during prolonged exercise. J Appl Physiol (1985) (1986) 61:1796–801. doi: 10.1152/JAPPL.1986.61.5.1796 3781988

[114] Zouhal H Jayavel A Parasuraman K Hayes LD Tourny C Rhibi F. Effects of exercise training on anabolic and catabolic hormones with advanced age: A systematic review. Sports Med (2022) 52:1353–68. doi: 10.1007/S40279-021-01612-9 PMC9124654 34936049

[115] Fathi R Ghanbari-Niaki A Kraemer RR Talebi-Garakani E Saghebjoo M. The effect of exercise intensity on plasma and tissue acyl ghrelin concentrations in fasted rats. Regul Pept (2010) 165:133–7. doi: 10.1016/J.REGPEP.2010.05.013 20542063

[116] Leidy HJ Gardner JK Frye BR Snook ML Schuchert MK Richard EL. Circulating ghrelin is sensitive to changes in body weight during a diet and exercise program in normal-weight young women. J Clin Endocrinol Metab (2004) 89:2659–64. doi: 10.1210/JC.2003-031471 15181038

[117] Mason C Xiao L Imayama I Duggan CR Campbell KL Kong A. The effects of separate and combined dietary weight loss and exercise on fasting ghrelin concentrations in overweight and obese women: a randomized controlled trial. Clin Endocrinol (Oxf) (2015) 82:369–76. doi: 10.1111/CEN.12483 PMC4221575 24796864

[118] Kraemer RR Castracane D. Effect of acute and chronic exercise on ghrelin and adipocytokines during pubertal development. Med Sport Sci (2010) 55:156–73. doi: 10.1159/000321979 20956867

[119] Bowyer KP Carson JA Davis JM Wang X. The influence of exercise training dose on fasting acylated ghrelin concentration in older women. J Behav Med (2019) 42:567–72. doi: 10.1007/S10865-018-9990-Z PMC6525072 30448936

[120] Markofski MM Carrillo AE Timmerman KL Jennings K Coen PM Pence BD. Exercise training modifies ghrelin and adiponectin concentrations and is related to inflammation in older adults. J Gerontol A Biol Sci Med Sci (2014) 69:675–81. doi: 10.1093/GERONA/GLT132 PMC4111637 24013674

[121] Kraemer RR Castracane VD. Exercise and humoral mediators of peripheral energy balance: Ghrelin and adiponectin. Exp Biol Med (2007) 232:184–94. 17259325

[122] Shiiya T Ueno H Toshinai K Kawagoe T Naito S Tobina T. Significant lowering of plasma ghrelin but not des-acyl ghrelin in response to acute exercise in men. Endocr J (2011) 58:335–42. doi: 10.1507/ENDOCRJ.K11E-021 21436599

[123] Pan W Yu Y Cain CM Nyberg F Couraud PO Kastin AJ. Permeation of growth hormone across the blood-brain barrier. Endocrinology (2005) 146:4898–904. doi: 10.1210/en.2005-0587 16099858

[124] Pardridge WM. Transport of insulin-related peptides and glucose across the blood-brain barrier. Ann N Y Acad Sci (1993) 692:126–37. doi: 10.1111/J.1749-6632.1993.TB26211.X 8215017

[125] Nyberg F Burman P. Growth hormone and its receptors in the central nervous system–location and functional significance. Horm Res (1996) 45:18–22. doi: 10.1159/000184753 8742113

[126] Haugland KG Olberg A Lande A Kjelstrup KB Brun VH. Hippocampal growth hormone modulates relational memory and the dendritic spine density in CA1. Learn Mem (2020) 27:33–44. doi: 10.1101/LM.050229.119 31949035 PMC6970428

[127] Russo VC Gluckman PD Feldman EL Werther GA. The insulin-like growth factor system and its pleiotropic functions in brain. Endocr Rev (2005) 26:916–43. doi: 10.1210/ER.2004-0024 16131630

[128] David Åberg N Lind J Isgaard J Georg Kuhn H. Peripheral growth hormone induces cell proliferation in the intact adult rat brain. Growth Horm IGF Res (2010) 20:264–9. doi: 10.1016/J.GHIR.2009.12.003 20106687

[129] Åberg ND Johansson I Åberg MAI Lind J Johansson UE Cooper-Kuhn CM. Peripheral administration of GH induces cell proliferation in the brain of adult hypophysectomized rats. J Endocrinol (2009) 201:141–50. doi: 10.1677/JOE-08-0495 19171566

[130] Martínez-Moreno CG Arámburo C. Growth hormone (GH) and synaptogenesis. Vitam Horm (2020) 114:91–123. doi: 10.1016/BS.VH.2020.04.001 32723552

[131] Arámburo C Alba-Betancourt C Luna M Harvey S. Expression and function of growth hormone in the nervous system: a brief review. Gen Comp Endocrinol (2014) 203:35–42. doi: 10.1016/J.YGCEN.2014.04.035 24837495

[132] Åberg MAI Åberg ND Hedbäcker H Oscarsson J Eriksson PS. Peripheral infusion of IGF-I selectively induces neurogenesis in the adult rat hippocampus. J Neurosci (2000) 20:2896–903. doi: 10.1523/JNEUROSCI.20-08-02896.2000 PMC6772218 10751442

[133] Lichtenwalner RJ Forbes ME Bennett SA Lynch CD Sonntag WE Riddle DR. Intracerebroventricular infusion of insulin-like growth factor-I ameliorates the age-related decline in hippocampal neurogenesis. Neuroscience (2001) 107:603–13. doi: 10.1016/S0306-4522(01)00378-5 11720784

[134] Carlson SW Saatman KE. Central infusion of insulin-like growth factor-1 increases hippocampal neurogenesis and improves neurobehavioral function after traumatic brain injury. J Neurotrauma (2018) 35:1467–80. doi: 10.1089/NEU.2017.5374 PMC5998830 29455576

[135] Shi L Linville MC Tucker EW Sonntag WE Brunso-Bechtold JK. Differential effects of aging and insulin-like growth factor-1 on synapses in CA1 of rat hippocampus. Cereb Cortex (2005) 15:571–7. doi: 10.1093/CERCOR/BHH158 15319312

[136] Zhang H Han M Zhang X Sun X Ling F. The effect and mechanism of growth hormone replacement on cognitive function in rats with traumatic brain injury. PloS One (2014) 9:e108518. doi: 10.1371/JOURNAL.PONE.0108518 25268832 PMC4182486

[137] Arjunan A Sah DK Woo M Song J. Identification of the molecular mechanism of insulin-like growth factor-1 (IGF-1): a promising therapeutic target for neurodegenerative diseases associated with metabolic syndrome. Cell Biosci (2023) 13:16. doi: 10.1186/S13578-023-00966-Z 36691085 PMC9872444

[138] Mir S Cai W Carlson SW Saatman KE Andres DA. IGF-1 mediated neurogenesis involves a novel RIT1/akt/sox2 cascade. Sci Rep (2017) 7:3283. doi: 10.1038/S41598-017-03641-9 28607354 PMC5468318

[139] Zuloaga R Fuentes EN Molina A Valdés JA. The cAMP Response Element Binding protein (CREB) is activated by Insulin-like Growth Factor-1 (IGF-1) and regulates myostatin gene expression in skeletal myoblast. Biochem Biophys Res Commun (2013) 440:258–64. doi: 10.1016/J.BBRC.2013.09.067 24064350

[140] Li E Chung H Kim Y Kim DH Ryu JH Sato T. Ghrelin directly stimulates adult hippocampal neurogenesis: implications for learning and memory. Endocr J (2013) 60:781–9. doi: 10.1507/ENDOCRJ.EJ13-0008 23411585

[141] Junnila RK List EO Berryman DE Murrey JW Kopchick JJ. The GH/IGF-1 axis in ageing and longevity. Nat Rev Endocrinol (2013) 9:366–76. doi: 10.1038/nrendo.2013.67 PMC4074016 23591370

[142] Deijen JB Arwert LI Drent ML. The GH/IGF-I axis and cognitive changes across a 4-year period in healthy adults. ISRN Endocrinol (2011) 2011:1–6. doi: 10.5402/2011/249421 PMC3262636 22363870

[143] Quik EH Conemans EB Valk GD Kenemans JL Koppeschaar HPF van Dam PS. Cognitive performance in older males is associated with growth hormone secretion. Neurobiol Aging (2012) 33:582–7. doi: 10.1016/J.NEUROBIOLAGING.2010.03.022 20483505

[144] Ong LK Chow WZ TeBay C Kluge M Pietrogrande G Zalewska K. Growth hormone improves cognitive function after experimental stroke. Stroke (2018) 49:1257–66. doi: 10.1161/STROKEAHA.117.020557 29636425

[145] Dauwan M Begemann MJH Slot MIE Lee EHM Scheltens P Sommer IEC. Physical exercise improves quality of life, depressive symptoms, and cognition across chronic brain disorders: a transdiagnostic systematic review and meta-analysis of randomized controlled trials. J Neurol (2021) 268:1222–46. doi: 10.1007/S00415-019-09493-9 PMC7990819 31414194

[146] Blackmore DG Steyn FJ Carlisle A O’Keeffe I Vien KY Zhou X. An exercise “sweet spot” reverses cognitive deficits of aging by growth-hormone-induced neurogenesis. iScience (2021) 24(11):103275. doi: 10.1016/J.ISCI.2021.103275 34761193 PMC8567379

[147] Huang HJ Chen XR Han QQ Wang J Pilot A Yu R. The protective effects of Ghrelin/GHSR on hippocampal neurogenesis in CUMS mice. Neuropharmacology (2019) 155:31–43. doi: 10.1016/J.NEUROPHARM.2019.05.013 31103617

[148] Cui J Shen Y Li R. Estrogen synthesis and signaling pathways during aging: from periphery to brain. Trends Mol Med (2013) 19:197–209. doi: 10.1016/J.MOLMED.2012.12.007 23348042 PMC3595330

[149] Almey A Milner TA Brake WG. Estrogen receptors in the central nervous system and their implication for dopamine-dependent cognition in females. Horm Behav (2015) 74:125–38. doi: 10.1016/J.YHBEH.2015.06.010 PMC4820286 26122294

[150] Cano Sokoloff N Misra M Ackerman KE. Exercise, training, and the hypothalamic-pituitary-gonadal axis in men and women. Front Horm Res (2016) 47:27–43. doi: 10.1159/000445154 27348623 PMC7043068

[151] Blair JA McGee H Bhatta S Palm R Casadesus G. Hypothalamic-pituitary-gonadal axis involvement in learning and memory and Alzheimer’s disease: more than “just” estrogen. Front Endocrinol (Lausanne) (2015) 6:45. doi: 10.3389/FENDO.2015.00045 25859241 PMC4373369

[152] Warfvinge K Krause DN Maddahi A Edvinsson JCA Edvinsson L Haanes KA. Estrogen receptors α, β and GPER in the CNS and trigeminal system - molecular and functional aspects. J Headache Pain (2020) 21:131. doi: 10.1186/S10194-020-01197-0 33167864 PMC7653779

[153] Bean LA Ianov L Foster TC. Estrogen receptors, the hippocampus, and memory. Neuroscientist (2014) 20:534–45. doi: 10.1177/1073858413519865 PMC4317255 24510074

[154] Kumar V Chambon P. The estrogen receptor binds tightly to its responsive element as a ligand-induced homodimer. Cell (1988) 55:145–56. doi: 10.1016/0092-8674(88)90017-7 3167974

[155] Nilsson S Mäkelä S Treuter E Tujague M Thomsen J Andersson G. Mechanisms of estrogen action. Physiol Rev (2001) 81:1535–65. doi: 10.1152/PHYSREV.2001.81.4.1535 11581496

[156] Edwards DP Boonyaratanakornkit V. Rapid extranuclear signaling by the estrogen receptor (ER): MNAR couples ER and Src to the MAP kinase signaling pathway. Mol Interv (2003) 3:12–5. doi: 10.1124/MI.3.1.12 14993433

[157] Bunt JC Bahr JM Bemben DA. Comparison of estradiol and testosterone levels during and immediately following prolonged exercise in moderately active and trained males and females. Endocr Res (1987) 13:157–72. doi: 10.3109/07435808709023670 3622406

[158] Gray AB Telford RD Weidemann MJ. Endocrine response to intense interval exercise. Eur J Appl Physiol Occup Physiol (1993) 66:366–71. doi: 10.1007/BF00237784 8495701

[159] Kraemer RR Johnson LG Haltom R Kraemer GR Gaines H Drapcho M. Effects of hormone replacement on growth hormone and prolactin exercise responses in postmenopausal women. J Appl Physiol (1985) (1998) 84:703–8. doi: 10.1152/JAPPL.1998.84.2.703 9475883

[160] Pietranera L Brocca ME Roig P Lima A Garcia-Segura LM De Nicola AF. Estrogens are neuroprotective factors for hypertensive encephalopathy. J Steroid Biochem Mol Biol (2015) 146:15–25. doi: 10.1016/J.JSBMB.2014.04.001 24736028

[161] Bustamante-Barrientos FA Méndez-Ruette M Ortloff A Luz-Crawford P Rivera FJ Figueroa CD. The impact of estrogen and estrogen-like molecules in neurogenesis and neurodegeneration: beneficial or harmful? Front Cell Neurosci (2021) 15:636176. doi: 10.3389/fncel.2021.636176 33762910 PMC7984366

[162] Brännvall K Korhonen L Lindholm D. Estrogen-receptor-dependent regulation of neural stem cell proliferation and differentiation. Mol Cell Neurosci (2002) 21:512–20. doi: 10.1006/mcne.2002.1194 12498791

[163] Jun MW Liu L Brinton RD. Estradiol-17beta-induced human neural progenitor cell proliferation is mediated by an estrogen receptor beta-phosphorylated extracellularly regulated kinase pathway. Endocrinology (2008) 149:208–18. doi: 10.1210/EN.2007-1155 PMC2734499 17962344

[164] Okada M Makino A Nakajima M Okuyama S Furukawa S Furukawa Y. Estrogen stimulates proliferation and differentiation of neural stem/progenitor cells through different signal transduction pathways. Int J Mol Sci (2010) 11:4114–23. doi: 10.3390/IJMS11104114 PMC2996786 21152324

[165] Scharfman HE MacLusky NJ. Estrogen and brain-derived neurotrophic factor (BDNF) in hippocampus: complexity of steroid hormone-growth factor interactions in the adult CNS. Front Neuroendocrinol (2006) 27:415–35. doi: 10.1016/J.YFRNE.2006.09.004 PMC1778460 17055560

[166] Wroolie TE Kenna HA Williams KE Rasgon NL. Cognitive effects of hormone therapy continuation or discontinuation in a sample of women at risk for Alzheimer disease. Am J Geriatric Psychiatry (2015) 23:1117–26. doi: 10.1016/j.jagp.2015.05.009 PMC4654994 26209223

[167] Brown CM Suzuki S Jelks KAB Wise PM. Estradiol is a potent protective, restorative, and trophic factor after brain injury. Semin Reprod Med (2009) 27:240–9. doi: 10.1055/S-0029-1216277 PMC2846418 19401955

[168] Engler-Chiurazzi EB Singh M Simpkins JW. From the 90’s to now: A brief historical perspective on more than two decades of estrogen neuroprotection. Brain Res (2016) 1633:96–100. doi: 10.1016/J.BRAINRES.2015.12.044 26740397 PMC4762740

[169] Spritzer MD Roy EA. Testosterone and adult neurogenesis. Biomolecules (2020) 10(2):225. doi: 10.3390/BIOM10020225 32028656 PMC7072323

[170] Dufau ML Winters CA Hattori M Aquilano D Barañao JLS Nozu K. Hormonal regulation of androgen production by the Leydig cell. J Steroid Biochem (1984) 20:161–73. doi: 10.1016/0022-4731(84)90203-6 6323862

[171] Bienenfeld A Azarchi S Lo Sicco K Marchbein S Shapiro J Nagler AR. Androgens in women: Androgen-mediated skin disease and patient evaluation. J Am Acad Dermatol (2019) 80:1497–506. doi: 10.1016/J.JAAD.2018.08.062 30312644

[172] Sato K Iemitsu M Aizawa K Ajisaka R. Testosterone and DHEA activate the glucose metabolism-related signaling pathway in skeletal muscle. Am J Physiol Endocrinol Metab (2008) 294(5):E961–68. doi: 10.1152/AJPENDO.00678.2007 18349113

[173] Lu NZ Wardell SE Burnstein KL Defranco D Fuller PJ Giguere V. International Union of Pharmacology. LXV. The pharmacology and classification of the nuclear receptor superfamily: glucocorticoid, mineralocorticoid, progesterone, and androgen receptors. Pharmacol Rev (2006) 58:782–97. doi: 10.1124/PR.58.4.9 17132855

[174] Mooradian AD Morley JE Korenman SG. Biological actions of androgens. Endocr Rev (1987) 8:1–28. doi: 10.1210/EDRV-8-1-1 3549275

[175] Simpson ER. Sources of estrogen and their importance. J Steroid Biochem Mol Biol (2003) 86:225–30. doi: 10.1016/S0960-0760(03)00360-1 14623515

[176] Kraemer RR Durand RJ Acevedo EO Johnson LG Synovitz LB Kraemer GR. Effects of high-intensity exercise on leptin and testosterone concentrations in well-trained males. Endocrine (2003) 21:261–5. doi: 10.1385/ENDO:21:3:261 14515011

[177] Kraemer RR Acevedo EO Synovitz LB Hebert EP Gimpel T Castracane VD. Leptin and steroid hormone responses to exercise in adolescent female runners over a 7-week season. Eur J Appl Physiol (2001) 86:85–91. doi: 10.1007/s004210100500 11820328

[178] Kraemer WJ Häkkinen K Newton RU McCormick M Nindl BC Volek JS. Acute hormonal responses to heavy resistance exercise in younger and older men. Eur J Appl Physiol Occup Physiol (1998) 77:206–11. doi: 10.1007/S004210050323 9535580

[179] Diotel N Charlier TD Lefebvre d’Hellencourt C Couret D Trudeau VL Nicolau JC. Steroid transport, local synthesis, and signaling within the brain: roles in neurogenesis, neuroprotection, and sexual behaviors. Front Neurosci (2018) 12:84. doi: 10.3389/FNINS.2018.00084 29515356 PMC5826223

[180] Pompili A Iorio C Gasbarri A. Effects of sex steroid hormones on memory. Acta Neurobiol Exp (Wars) (2020) 80:117–28. doi: 10.21307/ane-2020-012 32602853

[181] Beyenburg S Watzka M Clusmann H Blümcke I Bidlingmaier F Elger CE. Androgen receptor mRNA expression in the human hippocampus. Neurosci Lett (2000) 294:25–8. doi: 10.1016/S0304-3940(00)01542-1 11044578

[182] Okamoto M Hojo Y Inoue K Matsui T Kawato S McEwen BS. Mild exercise increases dihydrotestosterone in hippocampus providing evidence for androgenic mediation of neurogenesis. Proc Natl Acad Sci U.S.A. (2012) 109:13100–5. doi: 10.1073/PNAS.1210023109 PMC3420174 22807478

[183] Spritzer MD Galea LAM. Testosterone and dihydrotestosterone, but not estradiol, enhance survival of new hippocampal neurons in adult male rats. Dev Neurobiol (2007) 67:1321–33. doi: 10.1002/DNEU.20457 17638384

[184] Duarte-Guterman P Lieblich SE Wainwright SR Chow C Chaiton JA Watson NV. Androgens enhance adult hippocampal neurogenesis in males but not females in an age-dependent manner. Endocrinology (2019) 160:2128–36. doi: 10.1210/EN.2019-00114 PMC6736050 31219567

[185] Heberden C. Sex steroids and neurogenesis. Biochem Pharmacol (2017) 141:56–62. doi: 10.1016/J.BCP.2017.05.019 28571999

[186] Bimonte-Nelson HA Singleton RS Nelson ME Eckman CB Barber J Scott TY. Testosterone, but not nonaromatizable dihydrotestosterone, improves working memory and alters nerve growth factor levels in aged male rats. Exp Neurol (2003) 181:301–12. doi: 10.1016/S0014-4886(03)00061-X 12782002

[187] Wagner BA Braddick VC Batson CG Cullen BH Miller LE Spritzer MD. Effects of testosterone dose on spatial memory among castrated adult male rats. Psychoneuroendocrinology (2018) 89:120–30. doi: 10.1016/J.PSYNEUEN.2017.12.025 PMC5878712 29414025

[188] Vingren JL Kraemer WJ Ratamess NA Anderson JM Volek JS Maresh CM. Testosterone physiology in resistance exercise and training: the up-stream regulatory elements. Sports Med (2010) 40:1037–53. doi: 10.2165/11536910-000000000-00000 21058750

[189] Gabai G Mongillo P Giaretta E Marinelli L. Do dehydroepiandrosterone (DHEA) and its sulfate (DHEAS) play a role in the stress response in domestic animals? Front Vet Sci (2020) 7:588835. doi: 10.3389/FVETS.2020.588835 33195624 PMC7649144

[190] Brown GA Vukovich MD Sharp RL Reifenrath TA Parsons KA King DS. Effect of oral DHEA on serum testosterone and adaptations to resistance training in young men. J Appl Physiol (1985) (1999) 87:2274–83. doi: 10.1152/JAPPL.1999.87.6.2274 10601178

[191] Karishma KK Herbert J. Dehydroepiandrosterone (DHEA) stimulates neurogenesis in the hippocampus of the rat, promotes survival of newly formed neurons and prevents corticosterone-induced suppression. Eur J Neurosci (2002) 16:445–53. doi: 10.1046/J.1460-9568.2002.02099.X 12193187

[192] Compagnone NA Mellon SH. Dehydroepiandrosterone: a potential signalling molecule for neocortical organization during development. Proc Natl Acad Sci U.S.A. (1998) 95:4678–83. doi: 10.1073/PNAS.95.8.4678 PMC22550 9539798

[193] Maggio M De Vita F Fisichella A Colizzi E Provenzano S Lauretani F. DHEA and cognitive function in the elderly. J Steroid Biochem Mol Biol (2015) 145:281–92. doi: 10.1016/J.JSBMB.2014.03.014 24794824

[194] Stárka L Dušková M Hill M. Dehydroepiandrosterone: a neuroactive steroid. J Steroid Biochem Mol Biol (2015) 145:254–60. doi: 10.1016/J.JSBMB.2014.03.008 24704258

[195] Johnson LG Kraemer GR Kraemer RR Gaines HE Haltom R Castracane VD. Effects of estrogen replacement therapy on dehydroepiandrosterone, dehydroepiandrosterone sulfate, and cortisol responses to exercise in postmenopausal women. Fertil Steril (1997) 68:836–43. doi: 10.1016/S0015-0282(97)00369-5 9389812

[196] Sato K Iemitsu M Katayama K Ishida K Kanao Y Saito M. Responses of sex steroid hormones to different intensities of exercise in endurance athletes. Exp Physiol (2016) 101:168–75. doi: 10.1113/EP085361 26518151

[197] Rahmani A Shoae-Hassani A Keyhanvar P Kheradmand D Darbandi-Azar A. Dehydroepiandrosterone stimulates nerve growth factor and brain derived neurotrophic factor in cortical neurons. Adv Pharmacol Sci (2013) 2013:506191. doi: 10.1155/2013/506191 24381588 PMC3867952

[198] Boström P Wu J Jedrychowski MP Korde A Ye L Lo JC. A PGC1-α-dependent myokine that drives brown-fat-like development of white fat and thermogenesis. Nature (2012) 481:463–8. doi: 10.1038/NATURE10777 PMC3522098 22237023

[199] Jackson TC Gorse K Herrmann JR Kochanek PM. Hippocampal and prefrontal cortical brain tissue levels of irisin and GDF15 receptor subunits in children. Mol Neurobiol (2021) 58:2145–57. doi: 10.1007/S12035-020-02250-4 PMC7788542 33411243

[200] Stepto NK Benziane B Wadley GD Chibalin AV Canny BJ Eynon N. Short-term intensified cycle training alters acute and chronic responses of PGC1α and Cytochrome C oxidase IV to exercise in human skeletal muscle. PloS One (2012) 7(12):e53080. doi: 10.1371/JOURNAL.PONE.0053080 23285255 PMC3532354

[201] Parada-Sánchez SG Macias-Cervantes MH Pérezvázquez V Vargas-Ortiz K. The effects of different types of exercise on circulating irisin levels in healthy individuals and in people with overweight, metabolic syndrome and type 2 diabetes. Physiol Res (2022) 71:457–75. doi: 10.33549/PHYSIOLRES.934896 PMC9616589 35770469

[202] Qiu S Bosnyák E Treff G Steinacker JM Nieß AM Krüger K. Acute exercise-induced irisin release in healthy adults: Associations with training status and exercise mode. Eur J Sport Sci (2018) 18:1226–33. doi: 10.1080/17461391.2018.1478452 29848211

[203] Liu S Cui F Ning K Wang Z Fu P Wang D. Role of irisin in physiology and pathology. Front Endocrinol (Lausanne) (2022) 13:962968. doi: 10.3389/FENDO.2022.962968 36225200 PMC9549367

[204] Zhao J Su Z Qu C Dong Y. Effects of 12 weeks resistance training on serum irisin in older male adults. Front Physiol (2017) 8:171. doi: 10.3389/FPHYS.2017.00171 28382004 PMC5360699

[205] McCormick JJ King KE Notley SR Fujii N Boulay P Sigal RJ. Exercise in the heat induces similar elevations in serum irisin in young and older men despite lower resting irisin concentrations in older adults. J Therm Biol (2022) 104:103189. doi: 10.1016/J.JTHERBIO.2022.103189 35180967

[206] Jürimäe J Purge P Remmel L Ereline J Kums T Kamandulis S. Changes in irisin, inflammatory cytokines and aerobic capacity in response to three weeks of supervised sprint interval training in older men. J Sports Med Phys Fitness (2023) 63:162–9. doi: 10.23736/S0022-4707.22.13949-6 35686866

[207] Wrann CD White JP Salogiannnis J Laznik-Bogoslavski D Wu J Ma D. Exercise induces hippocampal BDNF through a PGC-1α/FNDC5 pathway. Cell Metab (2013) 18:649–59. doi: 10.1016/j.cmet.2013.09.008 PMC3980968 24120943

[208] Severinsen MCK Pedersen BK. Muscle-organ crosstalk: the emerging roles of myokines. Endocr Rev (2020) 41:594–609. doi: 10.1210/ENDREV/BNAA016 32393961 PMC7288608

[209] Lourenco MV de Freitas GB Raony Í Ferreira ST De Felice FG. Irisin stimulates protective signaling pathways in rat hippocampal neurons. Front Cell Neurosci (2022) 16:953991. doi: 10.3389/FNCEL.2022.953991 36187295 PMC9518673

[210] Wang H Xu J Lazarovici P Quirion R Zheng W. cAMP response element-binding protein (CREB): A possible signaling molecule link in the pathophysiology of schizophrenia. Front Mol Neurosci (2018) 11:255. doi: 10.3389/FNMOL.2018.00255 30214393 PMC6125665

[211] Salgado R Benoy I Bogers J Weytjens R Vermeulen P Dirix L. Platelets and vascular endothelial growth factor (VEGF): A morphological and functional study. Angiogenesis (2001) 4:37–43. doi: 10.1023/A:1016611230747 11824377

[212] Ferrara N Davis-Smyth T. The biology of vascular endothelial growth factor. Endocr Rev (1997) 18:4–25. doi: 10.1210/EDRV.18.1.0287 9034784

[213] Taylor CW Ingham SA Hunt JEA Martin NRW Pringle JSM Ferguson RA. Exercise duration-matched interval and continuous sprint cycling induce similar increases in AMPK phosphorylation, PGC-1α and VEGF mRNA expression in trained individuals. Eur J Appl Physiol (2016) 116:1445–54. doi: 10.1007/S00421-016-3402-2 PMC4943987 27251406

[214] Patterson SD Leggate M Nimmo MA Ferguson RA. Circulating hormone and cytokine response to low-load resistance training with blood flow restriction in older men. Eur J Appl Physiol (2013) 113:713–9. doi: 10.1007/S00421-012-2479-5 22922803

[215] Vidoni ED Perales J Alshehri M Giles AM Siengsukon CF Burns JM. Aerobic exercise sustains performance of instrumental activities of daily living in early-stage Alzheimer disease. J Geriatr Phys Ther (2019) 42:E129–34. doi: 10.1519/JPT.0000000000000172 PMC6023779 29286983

[216] Luttrell MJ Mardis BR Bock JM Iwamoto E Hanada S Ueda K. Effect of age and acute exercise on circulating angioregulatory factors. J Aging Phys Act (2021) 29:423–30. doi: 10.1123/JAPA.2020-0024 33091872

[217] Cho SY Roh HT. Effects of exercise training on neurotrophic factors and blood-brain barrier permeability in young-old and old-old women. Int J Environ Res Public Health (2022) 19(24):16896. doi: 10.3390/IJERPH192416896 36554777 PMC9778715

[218] Wittko-Schneider IM Schneider FT Plate KH. Brain homeostasis: VEGF receptor 1 and 2-two unequal brothers in mind. Cell Mol Life Sci (2013) 70(5):1705–25. doi: 10.1007/S00018-013-1279-3 PMC3632714 23475067

[219] Mayhan WG. VEGF increases permeability of the blood-brain barrier via a nitric oxide synthase/cGMP-dependent pathway. Am J Physiol (1999) 276: C1148–53. doi: 10.1152/AJPCELL.1999.276.5.C1148 10329964

[220] Rich B Scadeng M Yamaguchi M Wagner PD Breen EC. Skeletal myofiber vascular endothelial growth factor is required for the exercise training-induced increase in dentate gyrus neuronal precursor cells. J Physiol (2017) 595:5931–43. doi: 10.1113/JP273994 PMC5577548 28597506

[221] Ben-Zeev T Shoenfeld Y Hoffman JR. The effect of exercise on neurogenesis in the brain. Israel Med Assoc J (2022) 24:533–8. 35971998

[222] Sun J Sha B Zhou W Yang Y. VEGF-mediated angiogenesis stimulates neural stem cell proliferation and differentiation in the premature brain. Biochem Biophys Res Commun (2010) 394:146–52. doi: 10.1016/J.BBRC.2010.02.132 20188072

[223] Herran E Perez- Gonzalez R Igartua M Pedraz JL Carro E Hernandez RM. Enhanced hippocampal neurogenesis in APP/ps1 mouse model of Alzheimer’s disease after implantation of VEGF-loaded PLGA nanospheres. Curr Alzheimer Res (2015) 12:932–40. doi: 10.2174/1567205012666151027121622 26502822

[224] Smith KJ Bleyer AJ Little WC Sane DC. The cardiovascular effects of erythropoietin. Cardiovasc Res (2003) 59:538–48. doi: 10.1016/S0008-6363(03)00468-1 14499855

[225] Jelkman W. Erythropoietin. J Endocrinol Invest (2003) 26:832–7. doi: 10.1007/BF03345232 14964434

[226] Dziembowska I Wójcik M Bukowski J Żekanowska E. Physical training increases erythroferrone levels in men. Biol (Basel) (2021) 10(11):1215. doi: 10.3390/BIOLOGY10111215 PMC8614876 34827208

[227] Hernández CC Burgos CF Gajardo AH Silva-Grecchi T Gavilan J Toledo JR. Neuroprotective effects of erythropoietin on neurodegenerative and ischemic brain diseases: The role of erythropoietin receptor. Neural Regener Res (2017) 12:1381–9. doi: 10.4103/1673-5374.215240 PMC5649449 29089974

[228] Ribeiro F Ribeiro IP Gonçalves AC Alves AJ Melo E Fernandes R. Effects of resistance exercise on endothelial progenitor cell mobilization in women. Sci Rep (2017) 7:17880. doi: 10.1038/S41598-017-18156-6 29259281 PMC5736626

[229] Roecker L Kowoll R Fraszl W Battal K Brechtel L Brachmann S. Observation of serum erythropoietin concentrations in female athletes for up to eight days after a marathon run. Clin Lab (2006) 52:511–3. 17078478

[230] Tomczyk M Kortas J Flis D Kaczorowska-Hac B Grzybkowska A Borkowska A. Marathon run-induced changes in the erythropoietin-erythroferrone-hepcidin axis are iron dependent. Int J Environ Res Public Health (2020) 17(8):2781. doi: 10.3390/IJERPH17082781 32316587 PMC7216253

[231] Duca L Da Ponte A Cozzi M Carbone A Pomati M Nava I. Changes in erythropoiesis, iron metabolism and oxidative stress after half-marathon. Intern Emerg Med (2006) 1:30–4. doi: 10.1007/BF02934717 16941810

[232] Azadan RJ Agha NH Kunz HE Baker FL Mylabathula PL Ledoux TA. The effects of normoxic endurance exercise on erythropoietin (EPO) production and the impact of selective β1 and non-selective β1 + β2 adrenergic receptor blockade. Eur J Appl Physiol (2021) 121:1499–511. doi: 10.1007/S00421-020-04558-4 33646423

[233] Montero D Breenfeldt-Andersen A Oberholzer L Haider T Goetze JP Meinild-Lundby AK. Erythropoiesis with endurance training: dynamics and mechanisms. Am J Physiol Regul Integr Comp Physiol (2017) 312:R894–902. doi: 10.1152/AJPREGU.00012.2017 28381454

[234] Yatsutani H Mori H Ito H Hayashi N Girard O Goto K. Endocrine and metabolic responses to endurance exercise under hot and hypoxic conditions. Front Physiol (2020) 11:932. doi: 10.3389/FPHYS.2020.00932 32973541 PMC7466541

[235] Brines ML Ghezzi P Keenan S Agnello D De Lanerolle NC Cerami C. Erythropoietin crosses the blood-brain barrier to protect against experimental brain injury. Proc Natl Acad Sci U.S.A. (2000) 97:10526–31. doi: 10.1073/PNAS.97.19.10526 PMC27058 10984541

[236] Digicaylioglu M Bichet S Marti HH Wenger RH Rivas LA Bauer C. Localization of specific erythropoietin binding sites in defined areas of the mouse brain. Proc Natl Acad Sci U.S.A. (1995) 92:3717–20. doi: 10.1073/PNAS.92.9.3717 PMC42032 7731971

[237] Noguchi CT Asavaritikrai P Teng R Jia Y. Role of erythropoietin in the brain. Crit Rev Oncol Hematol (2007) 64:159–71. doi: 10.1016/J.CRITREVONC.2007.03.001 PMC2083122 17482474

[238] Ratilal BO Arroja MMC Rocha JPF Fernandes AMA Barateiro AP Brites DMTO. Neuroprotective effects of erythropoietin pretreatment in a rodent model of transient middle cerebral artery occlusion. J Neurosurg (2014) 121:55–62. doi: 10.3171/2014.2.JNS132197 24702327

[239] Noh MY Cho KA Kim H Kim SM Kim SH. Erythropoietin modulates the immune-inflammatory response of a SOD1(G93A) transgenic mouse model of amyotrophic lateral sclerosis (ALS). Neurosci Lett (2014) 574:53–8. doi: 10.1016/J.NEULET.2014.05.001 24820540

[240] Maurice T Mustafa MH Desrumaux C Keller E Naert G García-Barceló MDLC. Intranasal formulation of erythropoietin (EPO) showed potent protective activity against amyloid toxicity in the Aβ_25-35_ non-transgenic mouse model of Alzheimer’s disease. J Psychopharmacol (2013) 27:1044–57. doi: 10.1177/0269881113494939 23813967

[241] Shen J Wu Y Xu JY Zhang J Sinclair SH Yanoff M. ERK- and Akt-dependent neuroprotection by erythropoietin (EPO) against glyoxal-AGEs via modulation of Bcl-xL, Bax, and BAD. Invest Ophthalmol Vis Sci (2010) 51:35–46. doi: 10.1167/IOVS.09-3544 19628748

[242] Pham TND Ma W Miller D Kazakova L Benchimol S. Erythropoietin inhibits chemotherapy-induced cell death and promotes a senescence-like state in leukemia cells. Cell Death Dis (2019) 10:22. doi: 10.1038/S41419-018-1274-6 30622244 PMC6325163

[243] Lin Y Brown L Hedley DW Barber DL Benchimol S. The death-promoting activity of p53 can be inhibited by distinct signaling pathways. Blood (2002) 100:3990–4000. doi: 10.1182/BLOOD-2002-02-0504 12393587

[244] Reisi P Arabpoor Z Rashidi B Alaei H Salami M Hamidi G. Erythropoietin improves neuronal proliferation in dentate gyrus of hippocampal formation in an animal model of Alzheimer’s disease. Adv BioMed Res (2012) 1:50. doi: 10.4103/2277-9175.100157 23326781 PMC3544128

[245] Wakhloo D Scharkowski F Curto Y Javed Butt U Bansal V Steixner-Kumar AA. Functional hypoxia drives neuroplasticity and neurogenesis via brain erythropoietin. Nat Commun (2020) 11:1313. doi: 10.1038/S41467-020-15041-1 32152318 PMC7062779

[246] Lightman SL Birnie MT Conway-Campbell BL. Dynamics of ACTH and cortisol secretion and implications for disease. Endocr Rev (2020) 41:470–90. doi: 10.1210/ENDREV/BNAA002 PMC7240781 32060528

[247] Timmermans S Souffriau J Libert C. A general introduction to glucocorticoid biology. Front Immunol (2019) 10:1545. doi: 10.3389/FIMMU.2019.01545 31333672 PMC6621919

[248] Koning ASCAM Buurstede JC Van Weert LTCM Meijer OC. Glucocorticoid and mineralocorticoid receptors in the brain: A transcriptional perspective. J Endocr Soc (2019) 3:1917–30. doi: 10.1210/JS.2019-00158 PMC6777400 31598572

[249] Bermejo JL Valldecabres R Villarrasa-Sapiña I Monfort-Torres G Marco-Ahulló A Ribeiro Do Couto B. Increased cortisol levels caused by acute resistance physical exercise impair memory and learning ability. PeerJ (2022) 10:e13000. doi: 10.7717/PEERJ.13000 35345590 PMC8957269

[250] Kraemer RR Acevedo EO Synovitz LB Durand RJ Johnson LG Petrella E. Glucoregulatory endocrine responses to intermittent exercise of different intensities: Plasma changes in a pancreatic β-cell peptide, amylin. Metabolism (2002) 51:657–63. doi: 10.1053/meta.2002.32023 11979402

[251] Kraemer RR Castracane DV Francois M Ghanbari-Niaki A Sirikul B Valverde RA. Effects of prolonged exercise on agouti-related protein: a pilot study. Endocrine (2012) 42:436–41. doi: 10.1007/S12020-012-9663-6 22477065

[252] Hill EE Zack E Battaglini C Viru M Viru A Hackney AC. Exercise and circulating cortisol levels: the intensity threshold effect. J Endocrinol Invest (2008) 31:587–91. doi: 10.1007/BF03345606 18787373

[253] Kraemer RR Acevedo EO Dzewaltowski D Kilgore JL Kraemer GR Castracane VD. Effects of low-volume resistive exercise on beta-endorphin and cortisol concentrations. Int J Sports Med (1996) 17:12–6. doi: 10.1055/S-2007-972801 8775570

[254] Ponce P Del Arco A Loprinzi P. Physical activity versus psychological stress: effects on salivary cortisol and working memory performance. Medicina (Kaunas) (2019) 55(5):119. doi: 10.3390/MEDICINA55050119 31052284 PMC6572132

[255] Vajda M Vanderka M Buzgó G Sedliak M Kampmiller T. The effect of different training modalities on resting hormonal level in active young males. J Appl BioMed (2021) 19:83–90. doi: 10.32725/JAB.2021.008 34907707

[256] Saaltink DJ Vreugdenhil E. Stress, glucocorticoid receptors, and adult neurogenesis: a balance between excitation and inhibition? Cell Mol Life Sci (2014) 71:2499–515. doi: 10.1007/S00018-014-1568-5 PMC4055840 24522255

[257] Anacker C Zunszain PA Cattaneo A Carvalho LA Garabedian MJ Thuret S. Antidepressants increase human hippocampal neurogenesis by activating the glucocorticoid receptor. Mol Psychiatry (2011) 16:738–50. doi: 10.1038/MP.2011.26 PMC3121947 21483429

[258] Sato H Horikawa Y Iizuka K Sakurai N Tanaka T Shihara N. Large-scale analysis of glucocorticoid target genes in rat hypothalamus. J Neurochem (2008) 106:805–14. doi: 10.1111/J.1471-4159.2008.05489.X 18489715

[259] Anacker C Cattaneo A Musaelyan K Zunszain PA Horowitz M Molteni R. Role for the kinase SGK1 in stress, depression, and glucocorticoid effects on hippocampal neurogenesis. Proc Natl Acad Sci U.S.A. (2013) 110:8708–13. doi: 10.1073/PNAS.1300886110 PMC3666742 23650397

[260] Yu S Patchev AV Wu Y Lu J Holsboer F Zhang JZ. Depletion of the neural precursor cell pool by glucocorticoids. Ann Neurol (2010) 67:21–30. doi: 10.1002/ANA.21812 20186952

[261] Barbosa BJAP de Souza-Talarico JN Nitrini R Brucki SMD. Reader response: Circulating cortisol and cognitive and structural brain measures: The Framingham Heart Study. Neurology (2019) 93:685. doi: 10.1212/WNL.0000000000008257 31591177

